# Supplementary material for: Genome-Wide Classification and Evolutionary Analysis Reveal Diverged Patterns of Chalcone Isomerase in Plants
Source: Biomolecules. 2022 Jul 8;12(7):961. doi: 10.3390/biom12070961 (PMC9313115; doi:10.3390/biom12070961)
Supplement: Supplementary file 1 [file biomolecules-12-00961-s001.zip › Figure S2 20 putative conserved motifs were identified using MEME suite in this study.pdf]

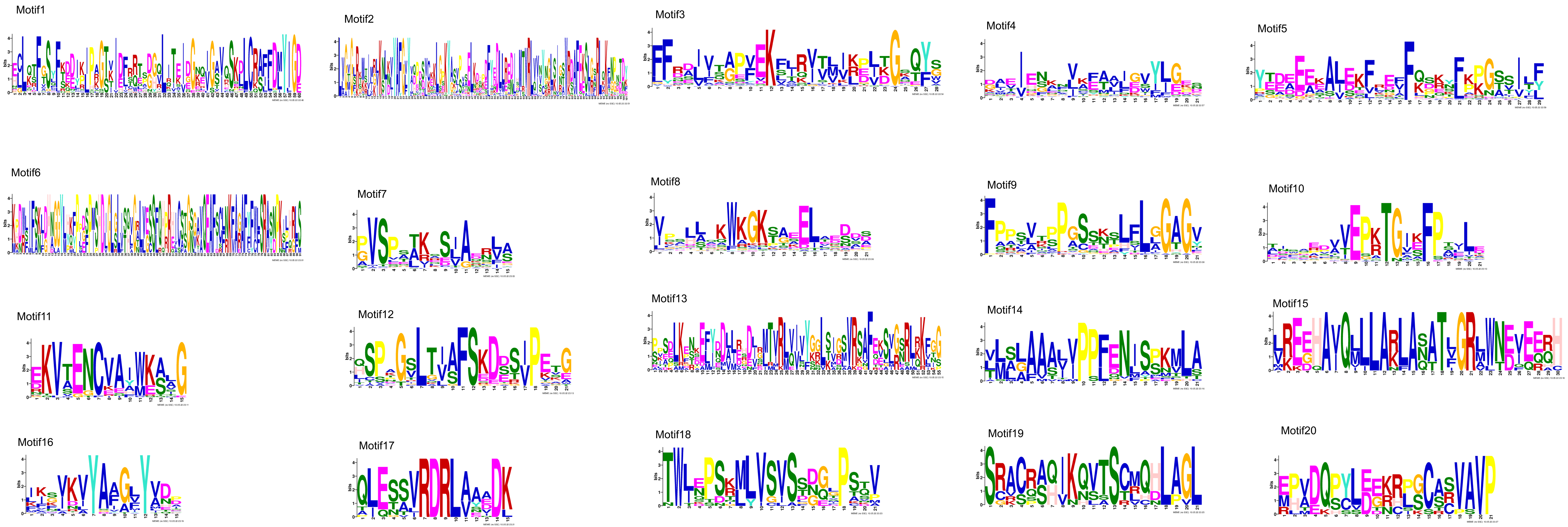
Motif1
Motif2
Motif3
Motif4
Motif5
Motif6
Motif7
Motif8
Motif9
Motif10
Motif11
Motif12
Motif13
Motif14
Motif15
Motif16
Motif17
Motif18
Motif19
Motif20
