# Supplementary material for: Genome-Wide Classification and Evolutionary Analysis Reveal Diverged Patterns of Chalcone Isomerase in Plants
Source: Biomolecules. 2022 Jul 8;12(7):961. doi: 10.3390/biom12070961 (PMC9313115; doi:10.3390/biom12070961)
Supplement: Supplementary file 1 [file biomolecules-12-00961-s001.zip › Figure S3 Protein sequence alignment of CHIs among 15 green plants.pdf]

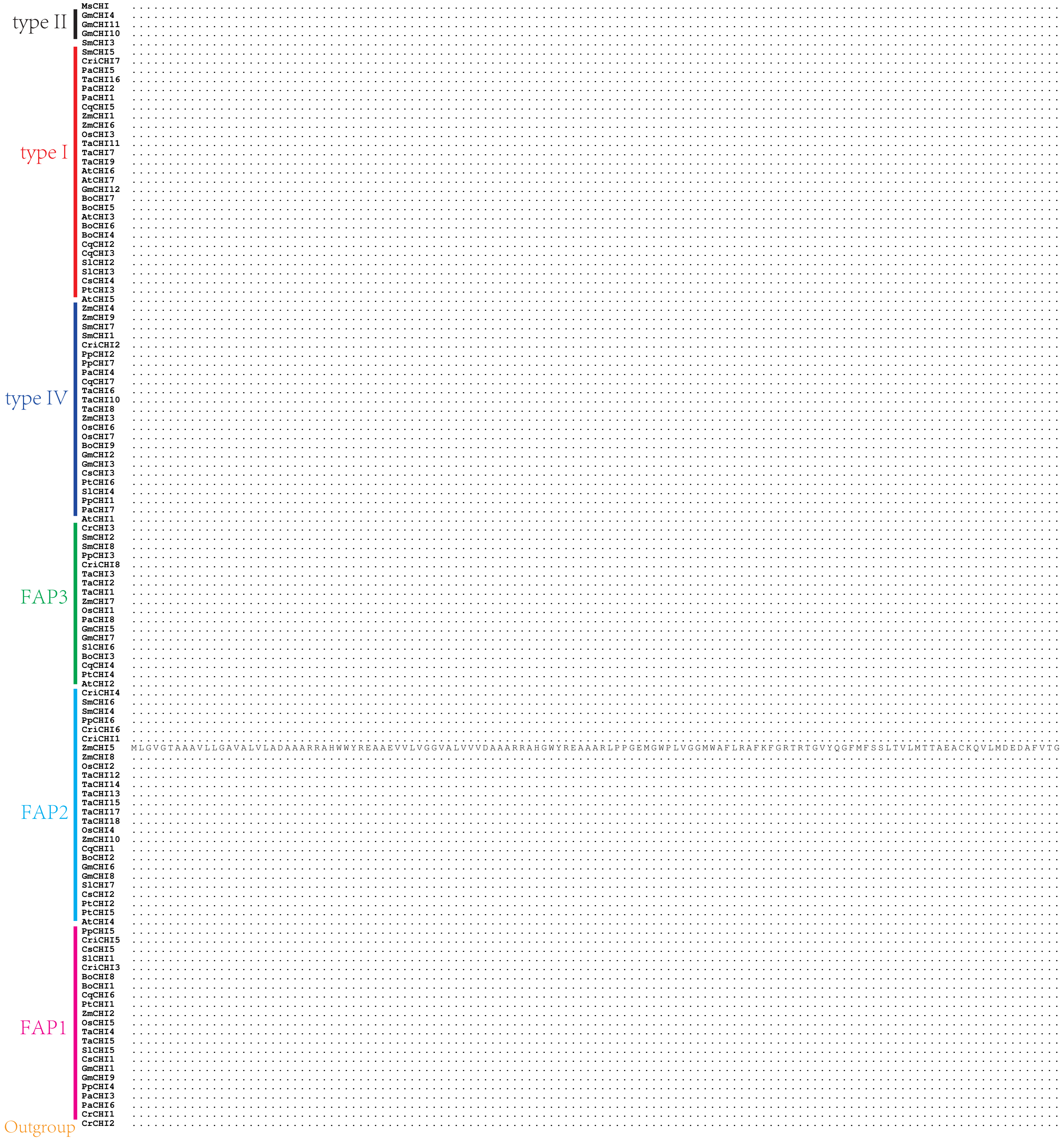
type II
MsCHI
GmCHI4
GmCHI11
GmCHI10
SmCHI3
type I
SmCHI5
CriCHI7
PaCHI5
TaCHI16
PaCHI2
PaCHI1
CqCHI5
ZmCHI1
ZmCHI6
OsCHI3
TaCHI11
TaCHI7
TaCHI9
AtCHI6
AtCHI7
GmCHI12
BoCHI7
BoCHI5
AtCHI3
BoCHI6
BoCHI4
CqCHI2
CqCHI3
SlCHI2
SlCHI3
CsCHI4
PtCHI3
AtCHI5
type IV
ZmCHI4
ZmCHI9
SmCHI7
SmCHI1
CriCHI2
PpCHI2
PpCHI7
PaCHI4
CqCHI7
TaCHI6
TaCHI10
TaCHI8
ZmCHI3
OsCHI6
OsCHI7
BoCHI9
GmCHI2
GmCHI3
CsCHI3
PtCHI6
SlCHI4
PpCHI1
PaCHI7
AtCHI1
FAP3
CrCHI3
SmCHI2
SmCHI8
PpCHI3
CriCHI8
TaCHI3
TaCHI2
TaCHI1
ZmCHI7
OsCHI1
PaCHI8
GmCHI5
GmCHI7
SlCHI6
BoCHI3
CqCHI4
PtCHI4
AtCHI2
FAP2
CriCHI4
SmCHI6
SmCHI4
PpCHI6
CriCHI6
CriCHI1
ZmCHI5 MLGVGTAAAVLLGAVALVLADAAARRAHWWYREAAEVVLVGGVALVVVDAAARRAHGWYREAAARLPPGEMGWPLVGGMWAFLRAFKFGRTRTGVYQGFMFSSLTVLMTTAEACKQVLMDEDAFVTG
ZmCHI8
OsCHI2
TaCHI12
TaCHI14
TaCHI13
TaCHI15
TaCHI17
TaCHI18
OsCHI4
ZmCHI10
CqCHI1
BoCHI2
GmCHI6
GmCHI8
SlCHI7
CsCHI2
PtCHI2
PtCHI5
AtCHI4
FAP1
PpCHI5
CriCHI5
CsCHI5
SlCHI1
CriCHI3
BoCHI8
BoCHI1
CqCHI6
PtCHI1
ZmCHI2
OsCHI5
TaCHI4
TaCHI5
SlCHI5
CsCHI1
GmCHI1
GmCHI9
PpCHI4
PaCHI3
PaCHI6
Outgroup
CrCHI1
CrCHI2

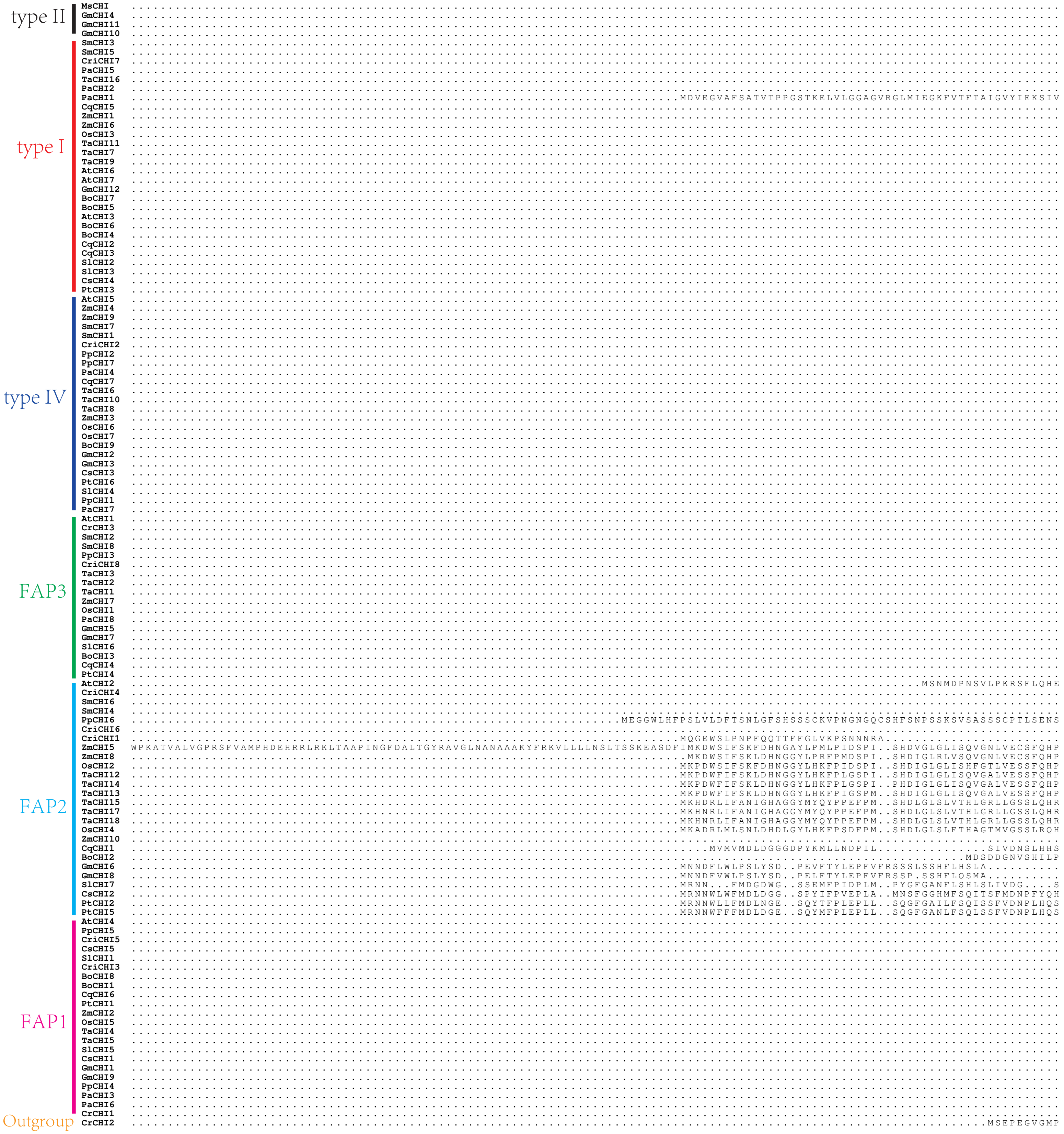
type II
type I
type IV
FAP3
FAP2
FAP1
Outgroup

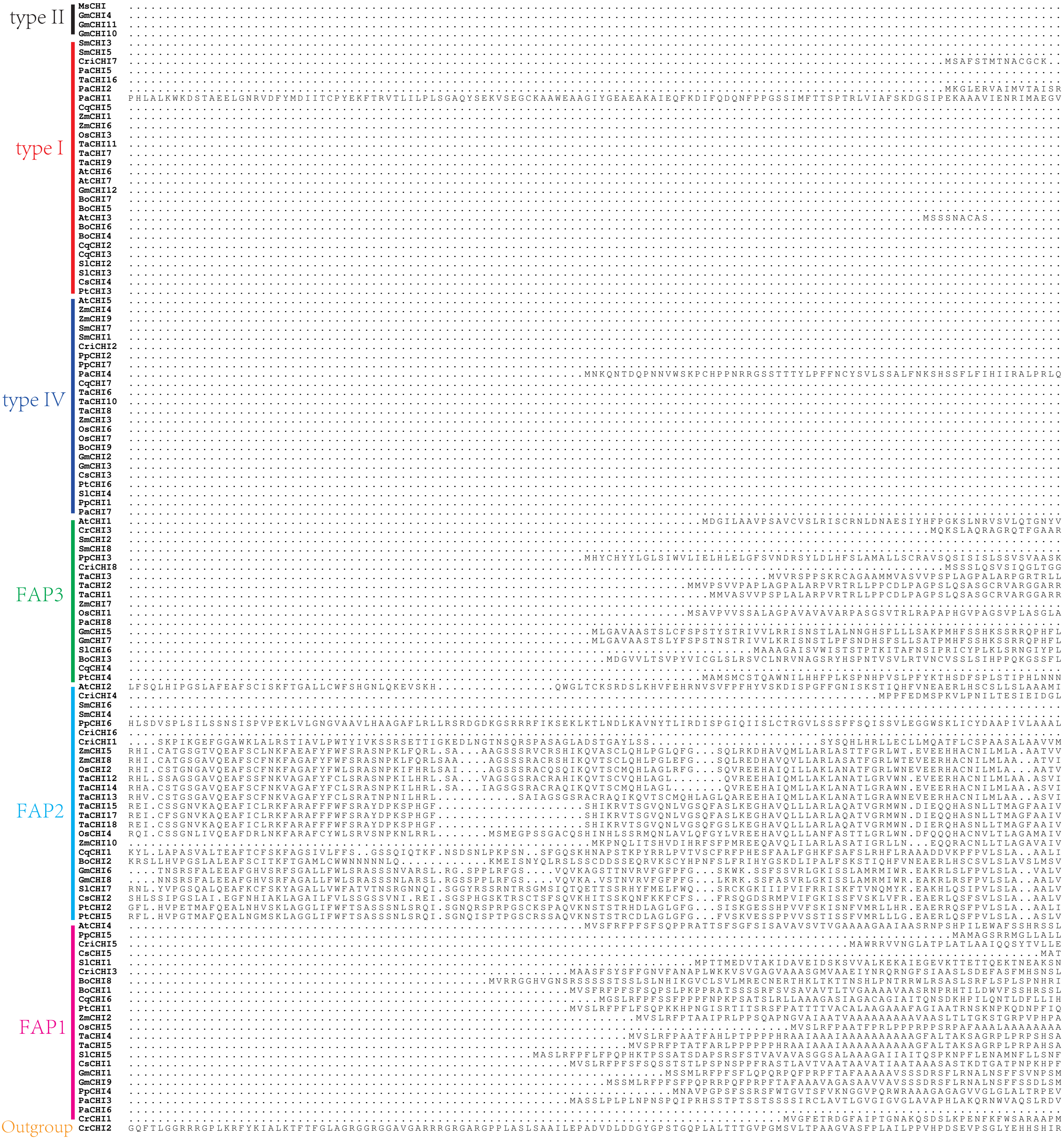

type II
type I
type IV
FAP3
FAP2
FAP1
Outgroup

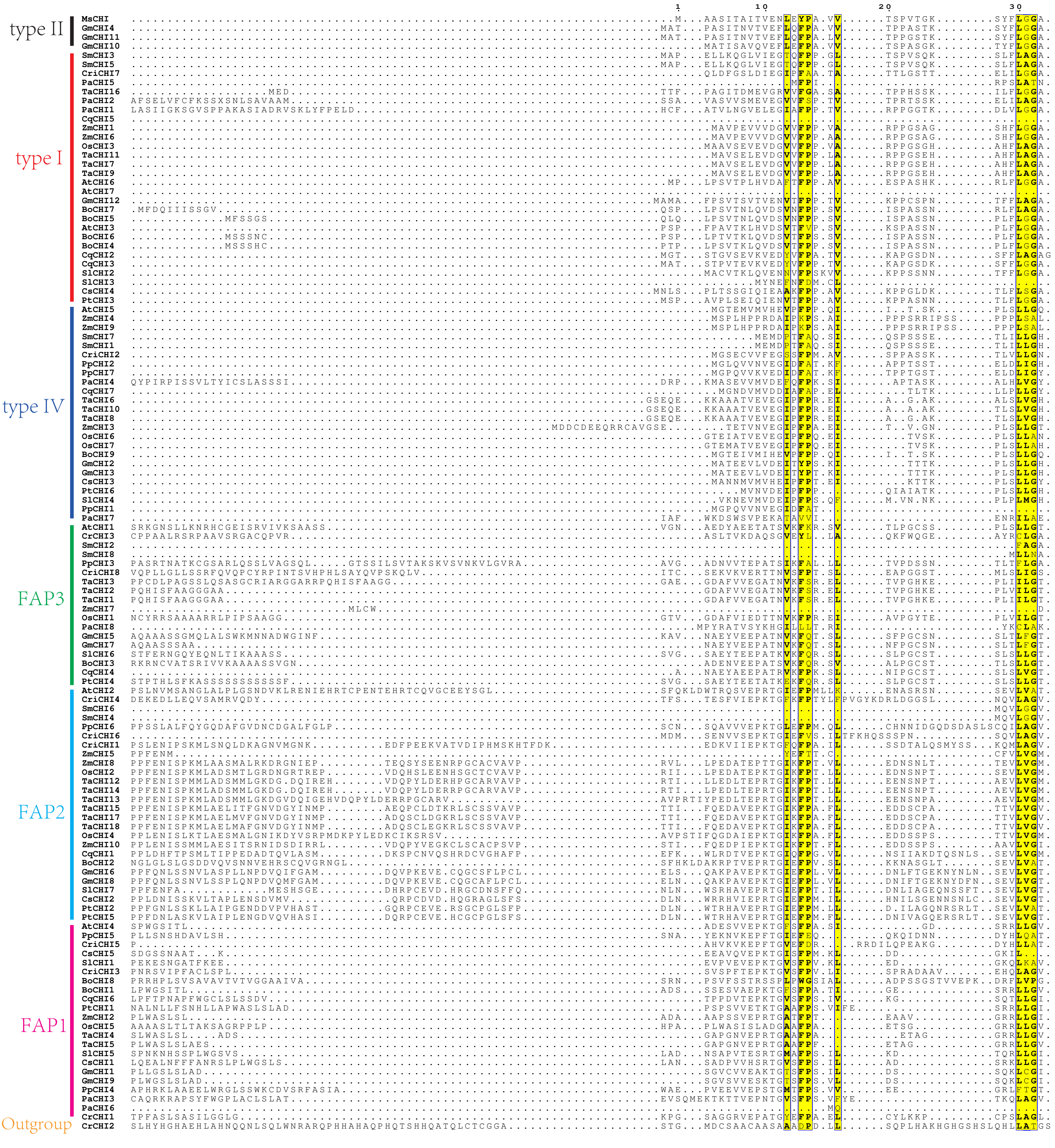
1
10
20
30
type II
MsCHI
GmCHI4
GmCHI11
GmCHI10
type I
SmCHI3
SmCHI5
CriCHI7
PaCHI5
TaCHI16
PaCHI2
PaCHI1
CqCHI5
ZmCHI1
ZmCHI6
OsCHI3
TaCHI11
TaCHI7
TaCHI9
AtCHI6
AtCHI7
GmCHI12
BoCHI7
BoCHI5
AtCHI3
BoCHI6
BoCHI4
CqCHI2
CqCHI3
SlCHI2
SlCHI3
CsCHI4
PtCHI3
type IV
AtCHI5
ZmCHI4
ZmCHI9
SmCHI7
SmCHI1
CriCHI2
PpCHI2
PpCHI7
PaCHI4
CqCHI7
TaCHI6
TaCHI10
TaCHI8
ZmCHI3
OsCHI6
OsCHI7
BoCHI9
GmCHI2
GmCHI3
CsCHI3
PtCHI6
SlCHI4
PpCHI1
PaCHI7
FAP3
AtCHI1
CrCHI3
SmCHI2
SmCHI8
PpCHI3
CriCHI8
TaCHI3
TaCHI2
TaCHI1
ZmCHI7
OsCHI1
PaCHI8
GmCHI5
GmCHI7
SlCHI6
BoCHI3
CqCHI4
PtCHI4
FAP2
AtCHI2
CriCHI4
SmCHI6
SmCHI4
PpCHI6
CriCHI6
CriCHI1
ZmCHI5
ZmCHI8
OsCHI2
TaCHI12
TaCHI14
TaCHI13
TaCHI15
TaCHI17
TaCHI18
OsCHI4
ZmCHI10
CqCHI1
BoCHI2
GmCHI6
GmCHI8
SlCHI7
CsCHI2
PtCHI2
PtCHI5
FAP1
AtCHI4
PpCHI5
CriCHI5
CsCHI5
SlCHI1
CriCHI3
BoCHI8
BoCHI1
CqCHI6
PtCHI1
ZmCHI2
OsCHI5
TaCHI4
TaCHI5
SlCHI5
CsCHI1
GmCHI1
GmCHI9
PpCHI4
PaCHI3
PaCHI6
Outgroup
CrCHI1
CrCHI2

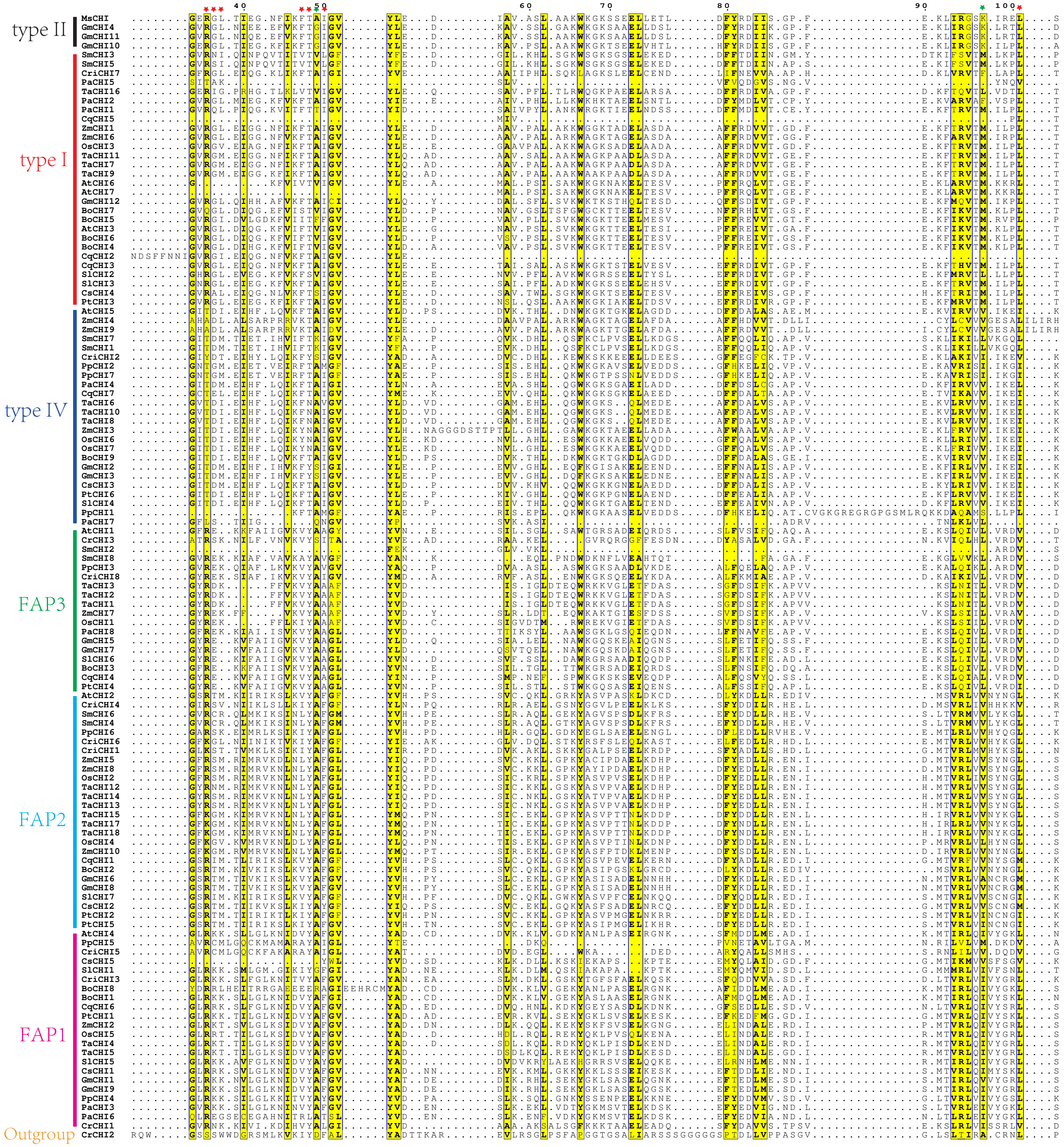
type II
type I
type IV
FAP3
FAP2
FAP1
Outgroup

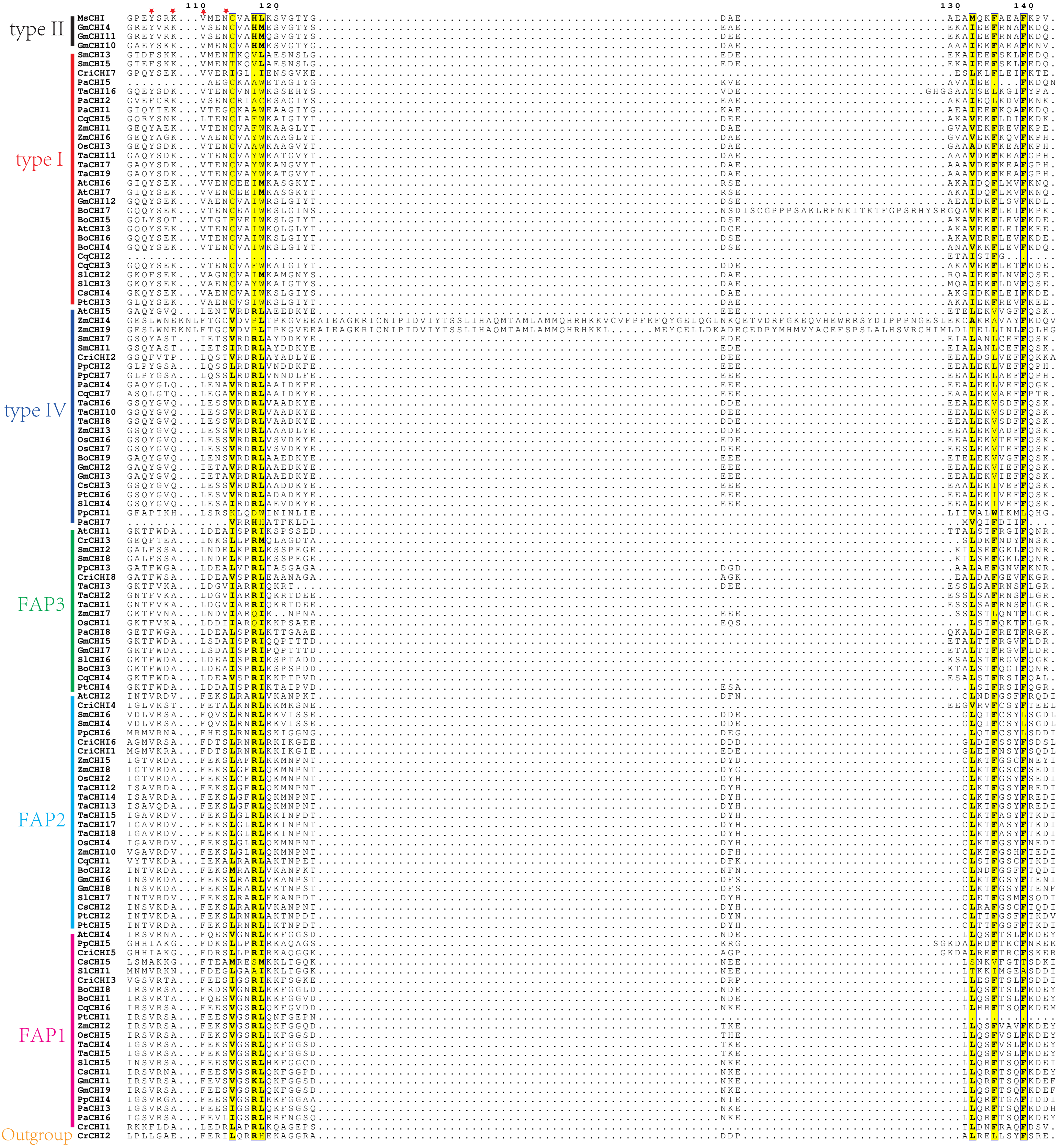

110
120
130
140
type II
type I
type IV
FAP3
FAP2
FAP1
Outgroup

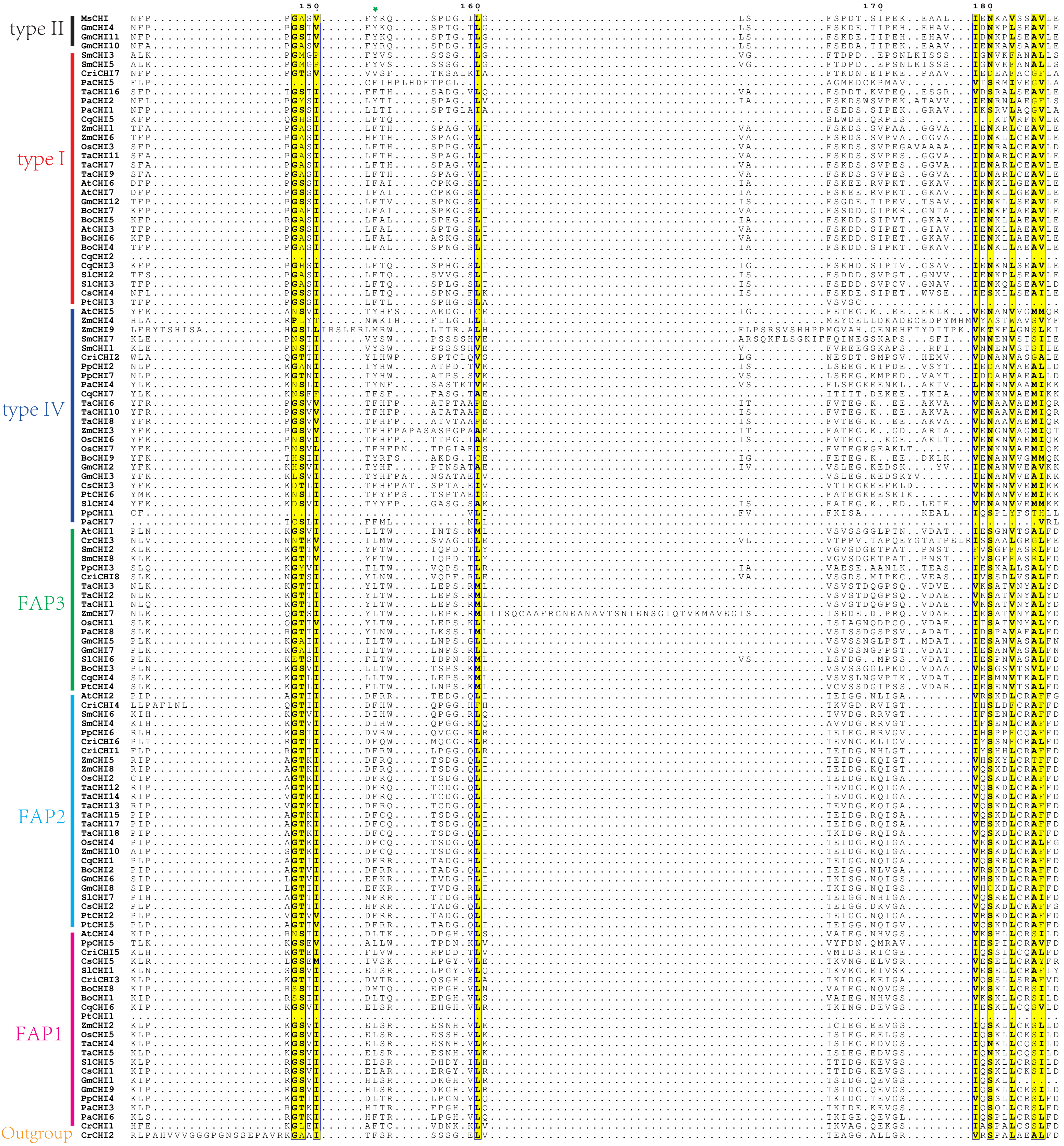

150
160
170
180
type II
type I
type IV
FAP3
FAP2
FAP1
Outgroup

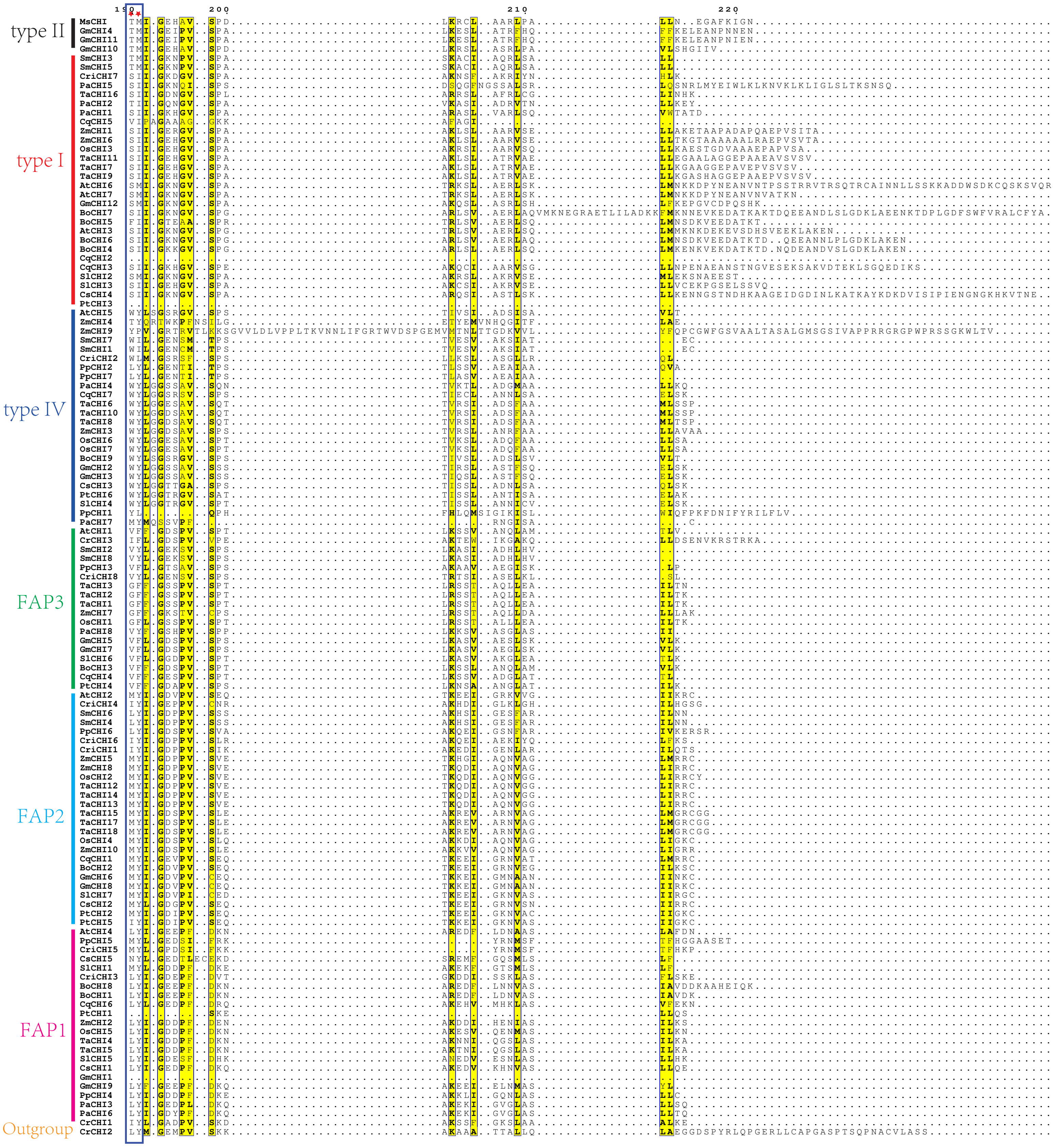

190
200
210
220
type II
type I
type IV
FAP3
FAP2
FAP1
Outgroup

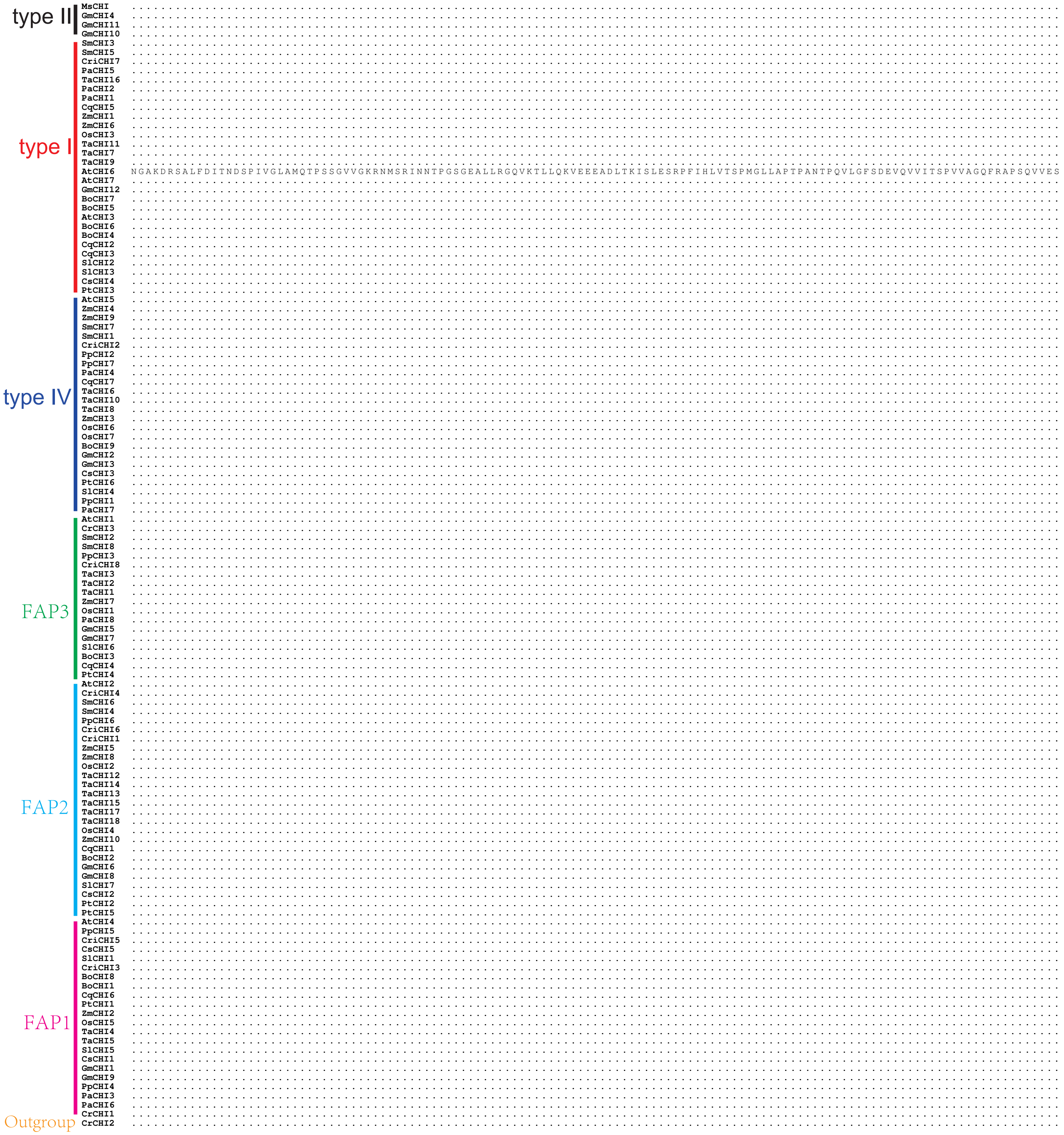
type II
MsCHI
GmCHI4
GmCHI11
GmCHI10
type I
SmCHI3
SmCHI5
CriCHI7
PaCHI5
TaCHI16
PaCHI2
PaCHI1
CqCHI5
ZmCHI1
ZmCHI6
OsCHI3
TaCHI11
TaCHI7
TaCHI9
AtCHI6
NGAKDRSALFDITNDSPIVGLAMQTPSSGVVGKRNMSRINNTPGSGEALLRGQVKTLLQKVEEEADLTKISLESRPFIHLVTSPMGLLAPTPANTPQVLGFSDEVQVVITSPVVAGQFRAPSQVVES
AtCHI7
GmCHI12
BoCHI7
BoCHI5
AtCHI3
BoCHI6
BoCHI4
CqCHI2
CqCHI3
SlCHI2
SlCHI3
CsCHI4
PtCHI3
type IV
AtCHI5
ZmCHI4
ZmCHI9
SmCHI7
SmCHI1
CriCHI2
PpCHI2
PpCHI7
PaCHI4
CqCHI7
TaCHI6
TaCHI10
TaCHI8
ZmCHI3
OsCHI6
OsCHI7
BoCHI9
GmCHI2
GmCHI3
CsCHI3
PtCHI6
SlCHI4
PpCHI1
PaCHI7
FAP3
AtCHI1
CrCHI3
SmCHI2
SmCHI8
PpCHI3
CriCHI8
TaCHI3
TaCHI2
TaCHI1
ZmCHI7
OsCHI1
PaCHI8
GmCHI5
GmCHI7
SlCHI6
BoCHI3
CqCHI4
PtCHI4
FAP2
AtCHI2
CriCHI4
SmCHI6
SmCHI4
PpCHI6
CriCHI6
CriCHI1
ZmCHI5
ZmCHI8
OsCHI2
TaCHI12
TaCHI14
TaCHI13
TaCHI15
TaCHI17
TaCHI18
OsCHI4
ZmCHI10
CqCHI1
BoCHI2
GmCHI6
GmCHI8
SlCHI7
CsCHI2
PtCHI2
PtCHI5
FAP1
AtCHI4
PpCHI5
CriCHI5
CsCHI5
SlCHI1
CriCHI3
BoCHI8
BoCHI1
CqCHI6
PtCHI1
ZmCHI2
OsCHI5
TaCHI4
TaCHI5
SlCHI5
CsCHI1
GmCHI1
GmCHI9
PpCHI4
PaCHI3
PaCHI6
Outgroup
CrCHI1
CrCHI2

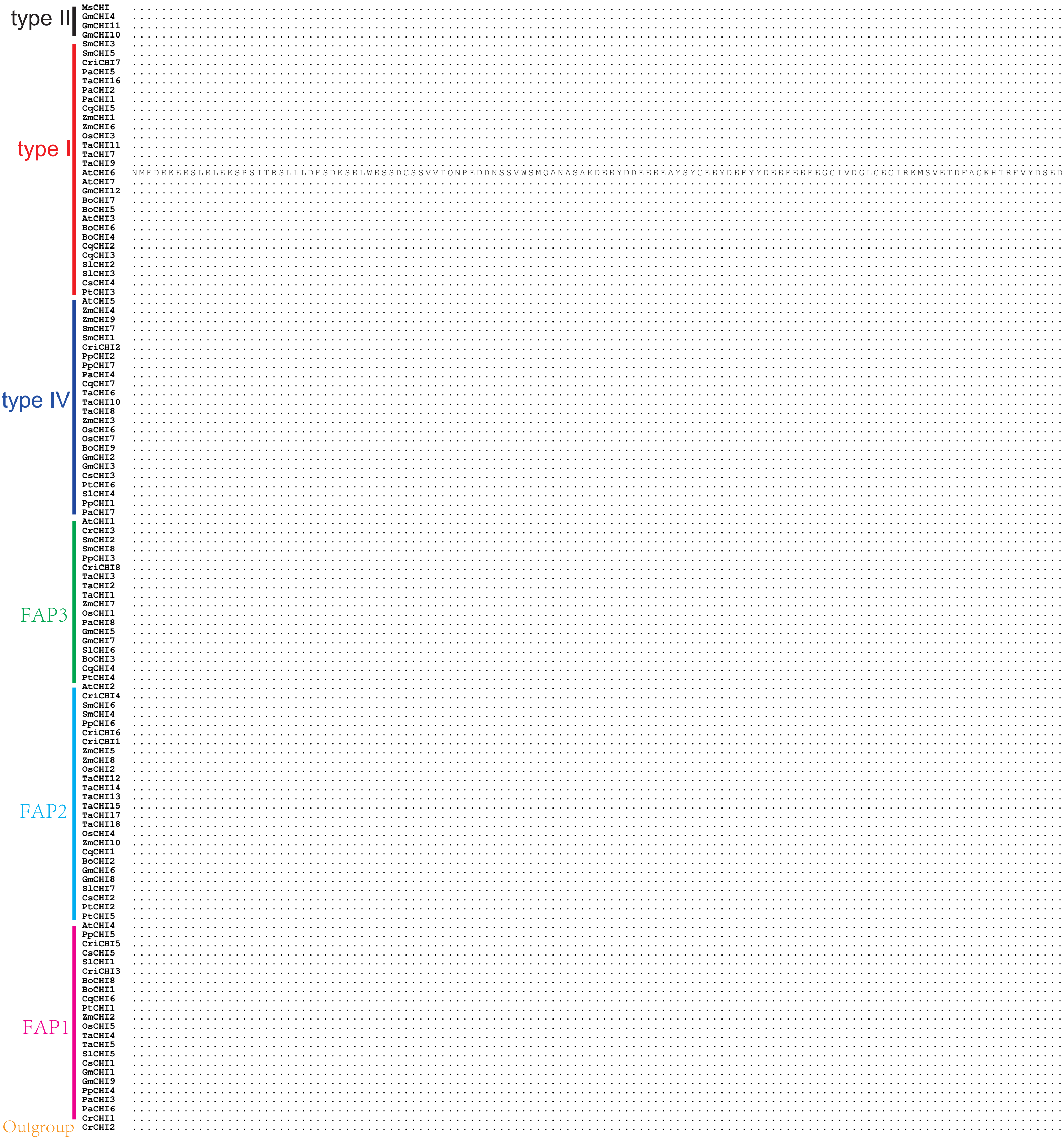

type II
MsCHI
GmCHI4
GmCHI11
GmCHI10
type I
SmCHI3
SmCHI5
CriCHI7
PaCHI5
TaCHI16
PaCHI2
PaCHI1
CqCHI5
ZmCHI1
ZmCHI6
OsCHI3
TaCHI11
TaCHI7
TaCHI9
AtCHI6 NMFDEKEESLELEKSPSITRSLLLDFSDKSELWESSDCSSVVTQNPEDDNSSVWSMQANASAKDEEYDDEEEEAYSYGEEYDEEYYDEEEEEEEGGIVDGLCEGIRKMSVETDFAGKHTRFVYDSED
AtCHI7
GmCHI12
BoCHI7
BoCHI5
AtCHI3
BoCHI6
BoCHI4
CqCHI2
CqCHI3
SlCHI2
SlCHI3
CsCHI4
PtCHI3
type IV
AtCHI5
ZmCHI4
ZmCHI9
SmCHI7
SmCHI1
CriCHI2
PpCHI2
PpCHI7
PaCHI4
CqCHI7
TaCHI6
TaCHI10
TaCHI8
ZmCHI3
OsCHI6
OsCHI7
BoCHI9
GmCHI2
GmCHI3
CsCHI3
PtCHI6
SlCHI4
PpCHI1
PaCHI7
FAP3
AtCHI1
CrCHI3
SmCHI2
SmCHI8
PpCHI3
CriCHI8
TaCHI3
TaCHI2
TaCHI1
ZmCHI7
OsCHI1
PaCHI8
GmCHI5
GmCHI7
SlCHI6
BoCHI3
CqCHI4
PtCHI4
FAP2
AtCHI2
CriCHI4
SmCHI6
SmCHI4
PpCHI6
CriCHI6
CriCHI1
ZmCHI5
ZmCHI8
OsCHI2
TaCHI12
TaCHI14
TaCHI13
TaCHI15
TaCHI17
TaCHI18
OsCHI4
ZmCHI10
CqCHI1
BoCHI2
GmCHI6
GmCHI8
SlCHI7
CsCHI2
PtCHI2
PtCHI5
FAP1
AtCHI4
PpCHI5
CriCHI5
CsCHI5
SlCHI1
CriCHI3
BoCHI8
BoCHI1
CqCHI6
PtCHI1
ZmCHI2
OsCHI5
TaCHI4
TaCHI5
SlCHI5
CsCHI1
GmCHI1
GmCHI9
PpCHI4
PaCHI3
PaCHI6
Outgroup
CrCHI1
CrCHI2

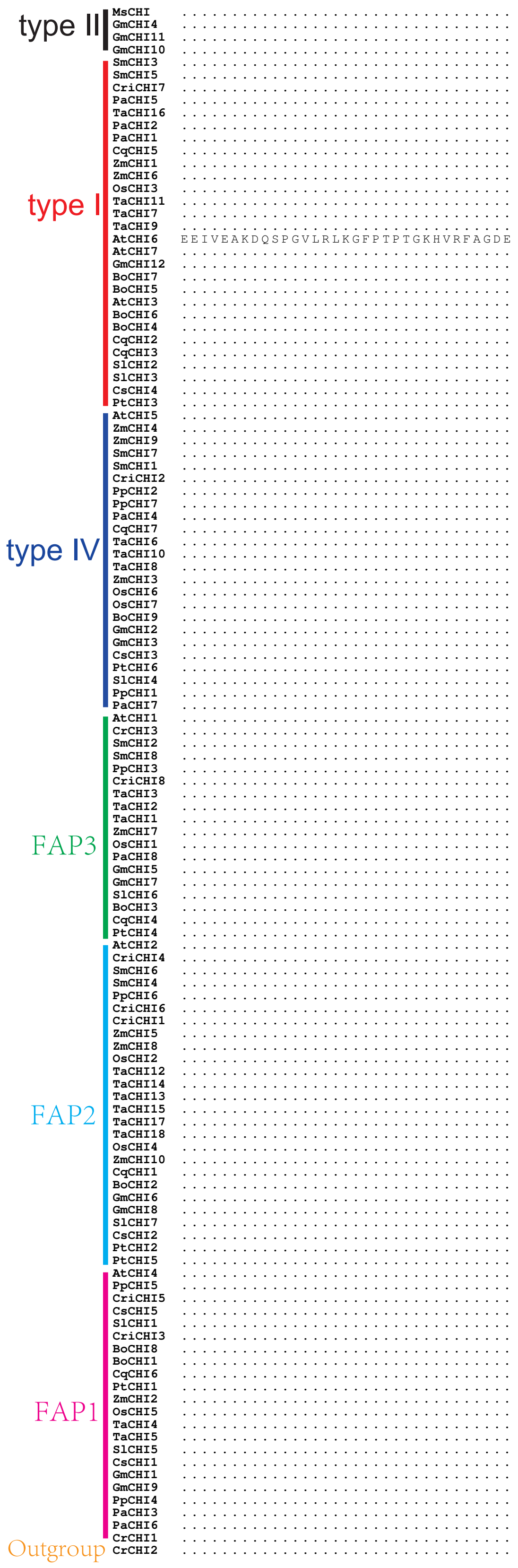

Supplementary Figure S3. Protein sequence alignment of CHIs among 15 green plants. The first line indicated the model protein sequence of MsCHI. The critical catalytic residues essential for *bona fide* CHI active sites are shown with red asterisks. Residues for the hydrogen bond network are marked with green asterisks. The blue boxes indicated the residues responsible for substrate preference.
