# Supplementary material for: Genome-Wide Classification and Evolutionary Analysis Reveal Diverged Patterns of Chalcone Isomerase in Plants
Source: Biomolecules. 2022 Jul 8;12(7):961. doi: 10.3390/biom12070961 (PMC9313115; doi:10.3390/biom12070961)
Supplement: Supplementary file 1 [file biomolecules-12-00961-s001.zip › Figure S4 3D homology model of 122 CHIs among 15 green plants.pdf]

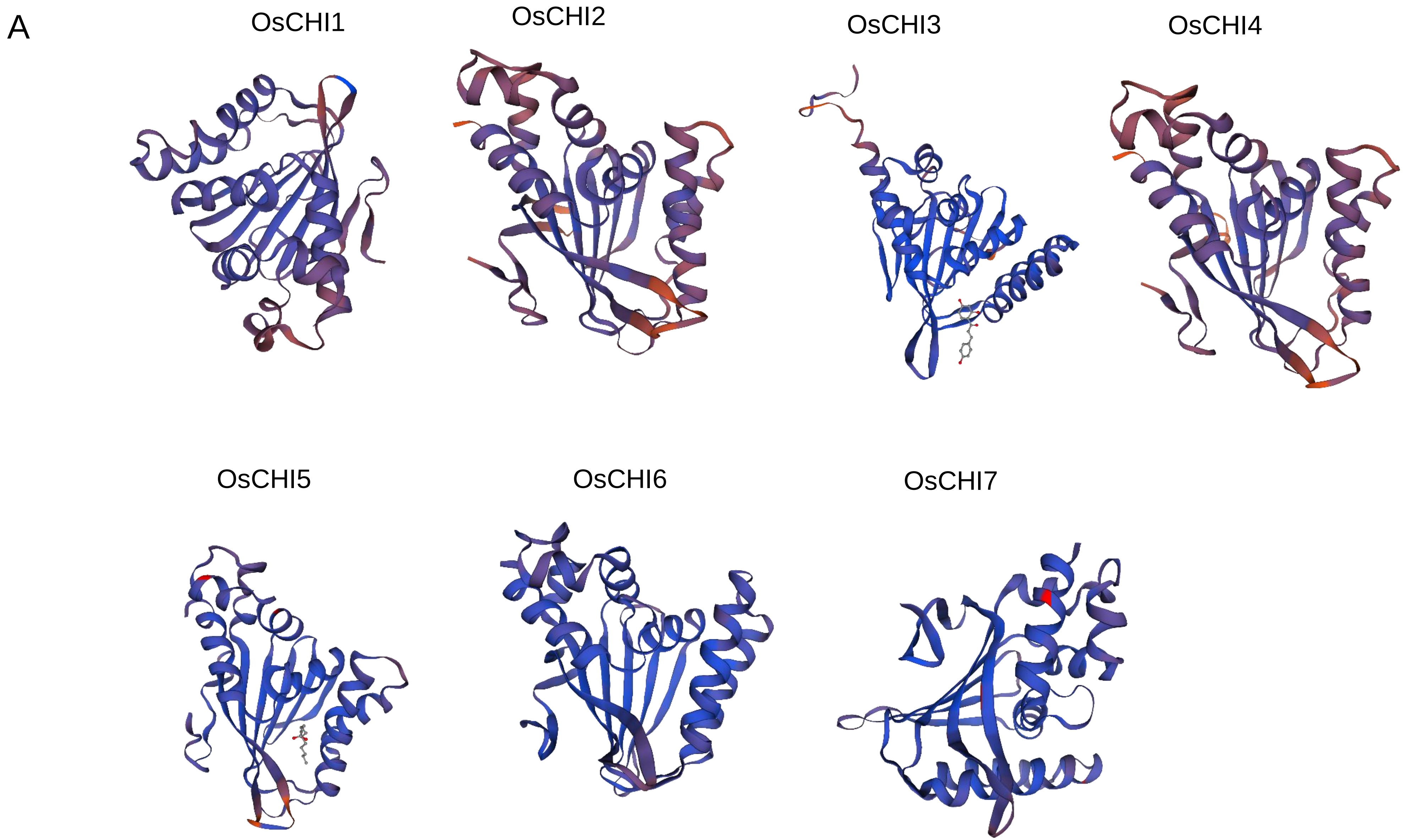
A
OsCHI1
OsCHI2
OsCHI3
OsCHI4
OsCHI5
OsCHI6
OsCHI7

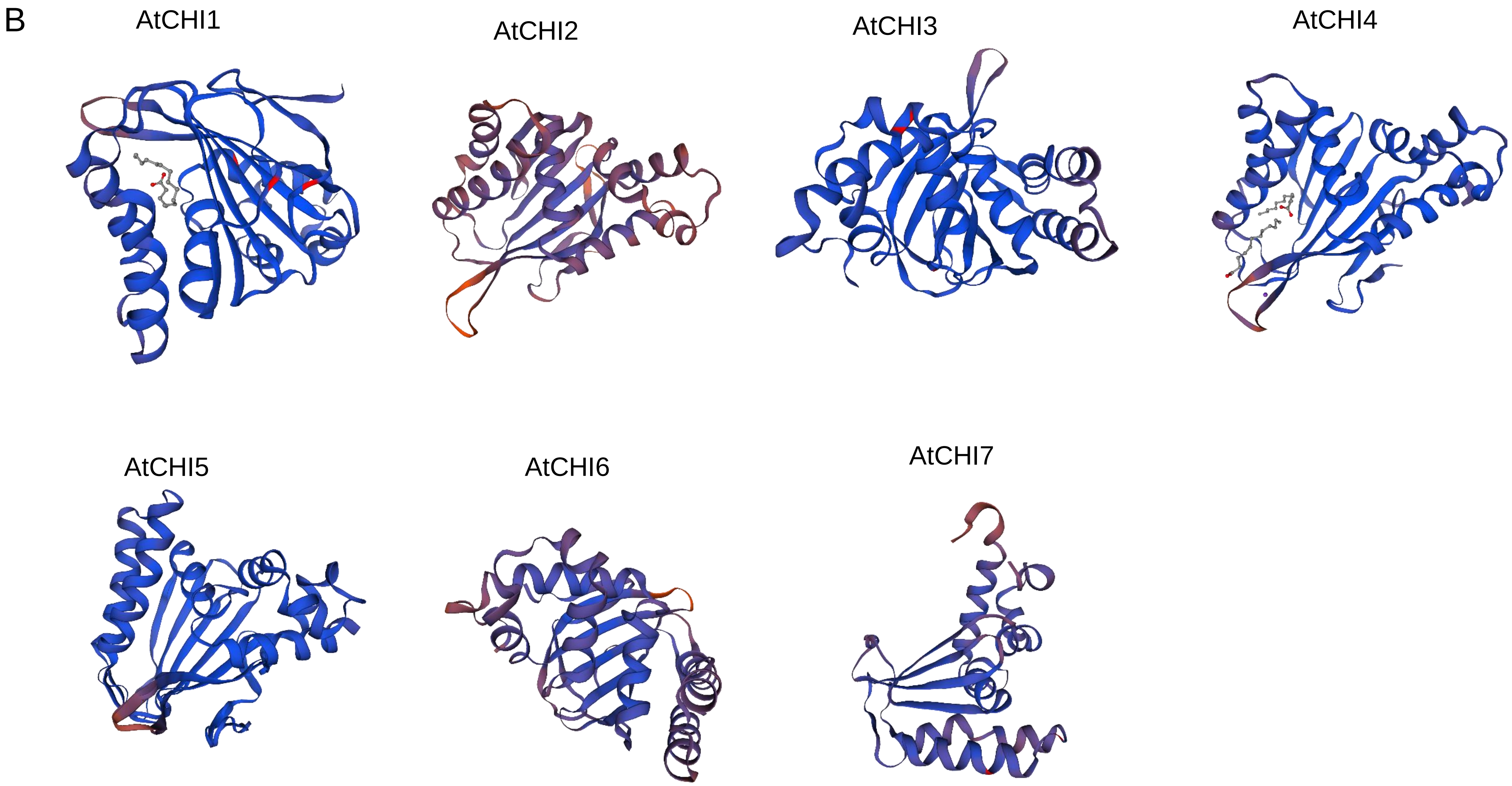
B
AtCHI1
AtCHI2
AtCHI3
AtCHI4
AtCHI5
AtCHI6
AtCHI7

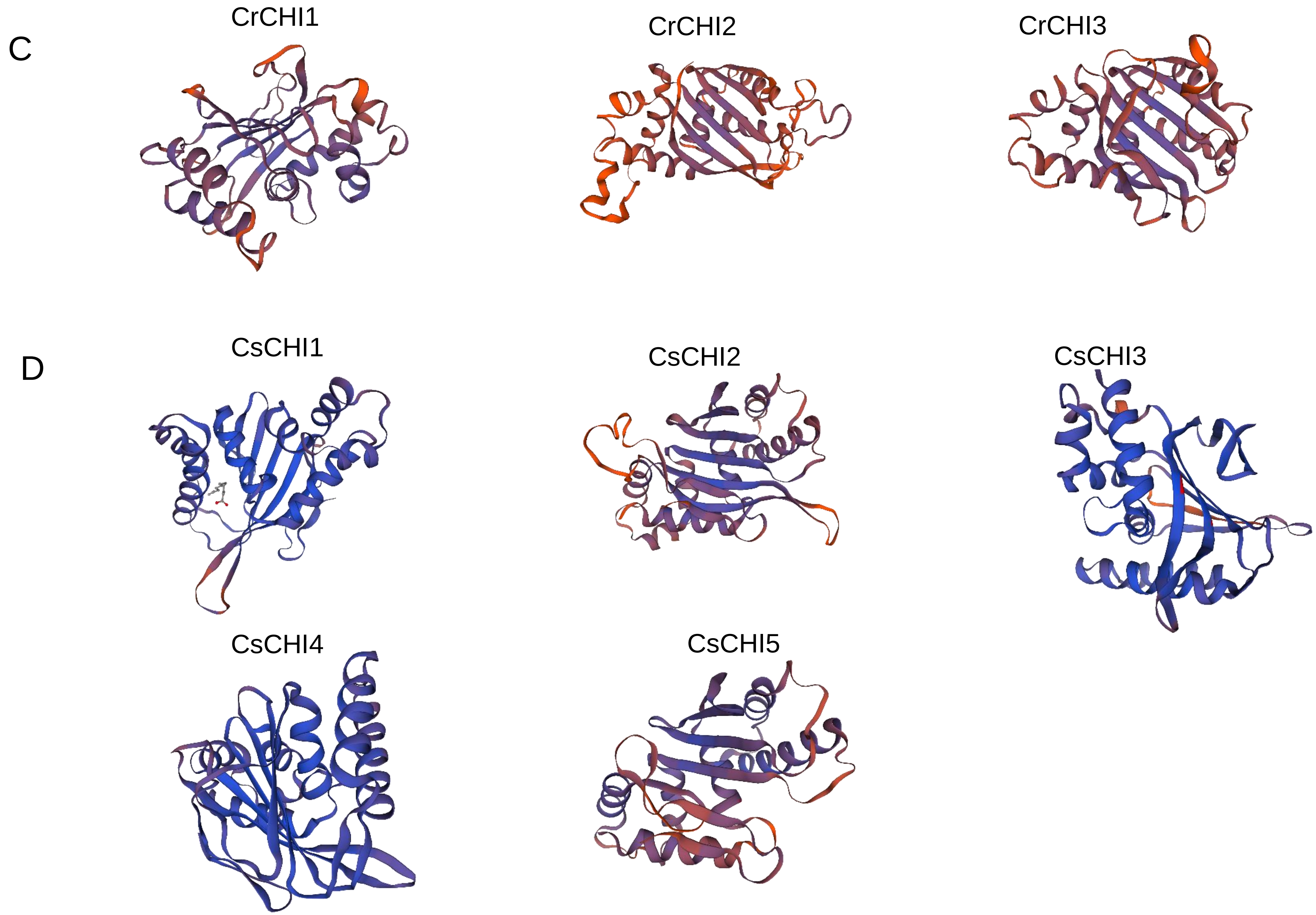
C
CrCHI1
CrCHI2
CrCHI3
D
CsCHI1
CsCHI2
CsCHI3
CsCHI4
CsCHI5

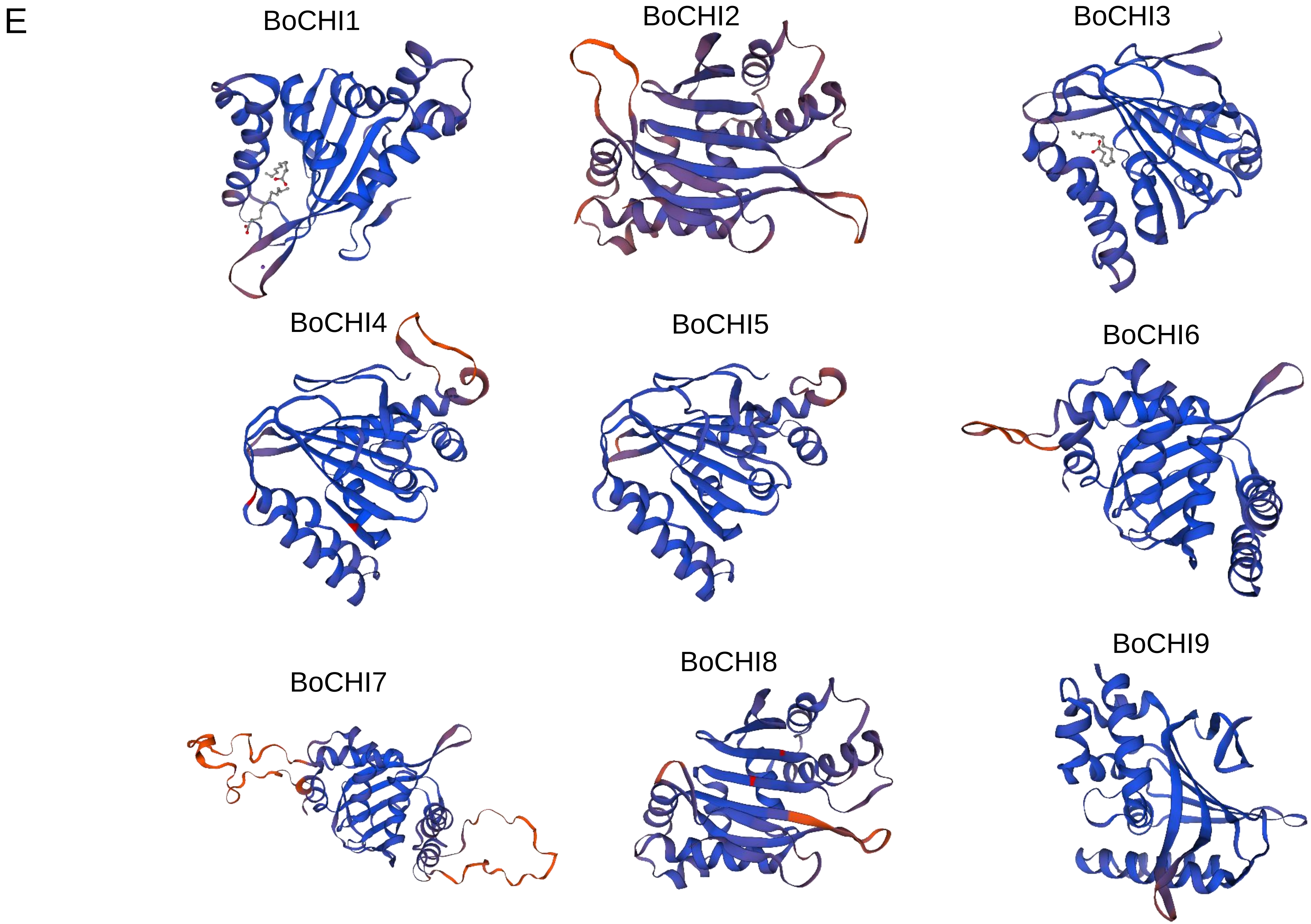
E
BoCHI1
BoCHI2
BoCHI3
BoCHI4
BoCHI5
BoCHI6
BoCHI7
BoCHI8
BoCHI9

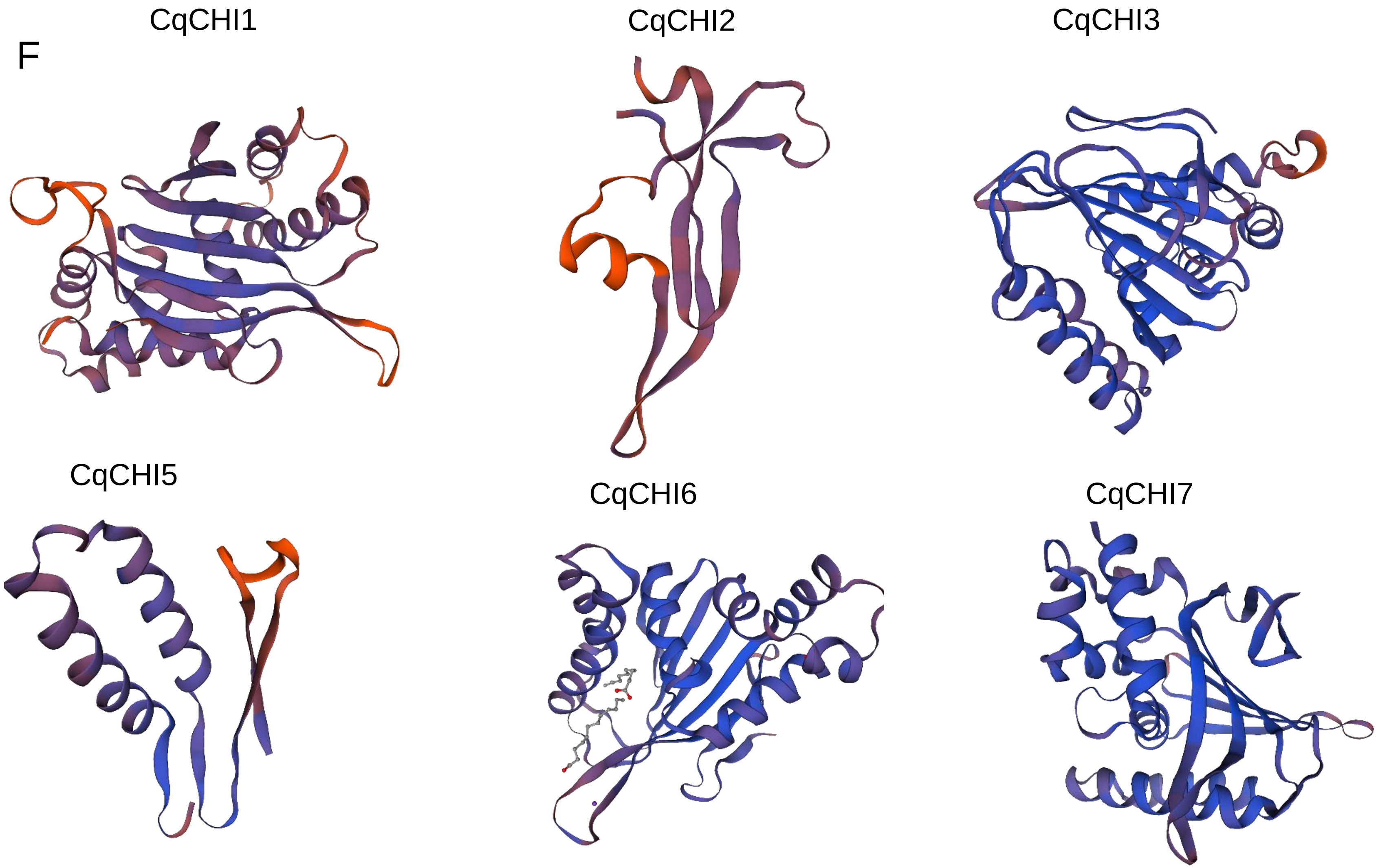
F
CqCHI1
CqCHI2
CqCHI3
CqCHI4
CqCHI5
CqCHI6
CqCHI7

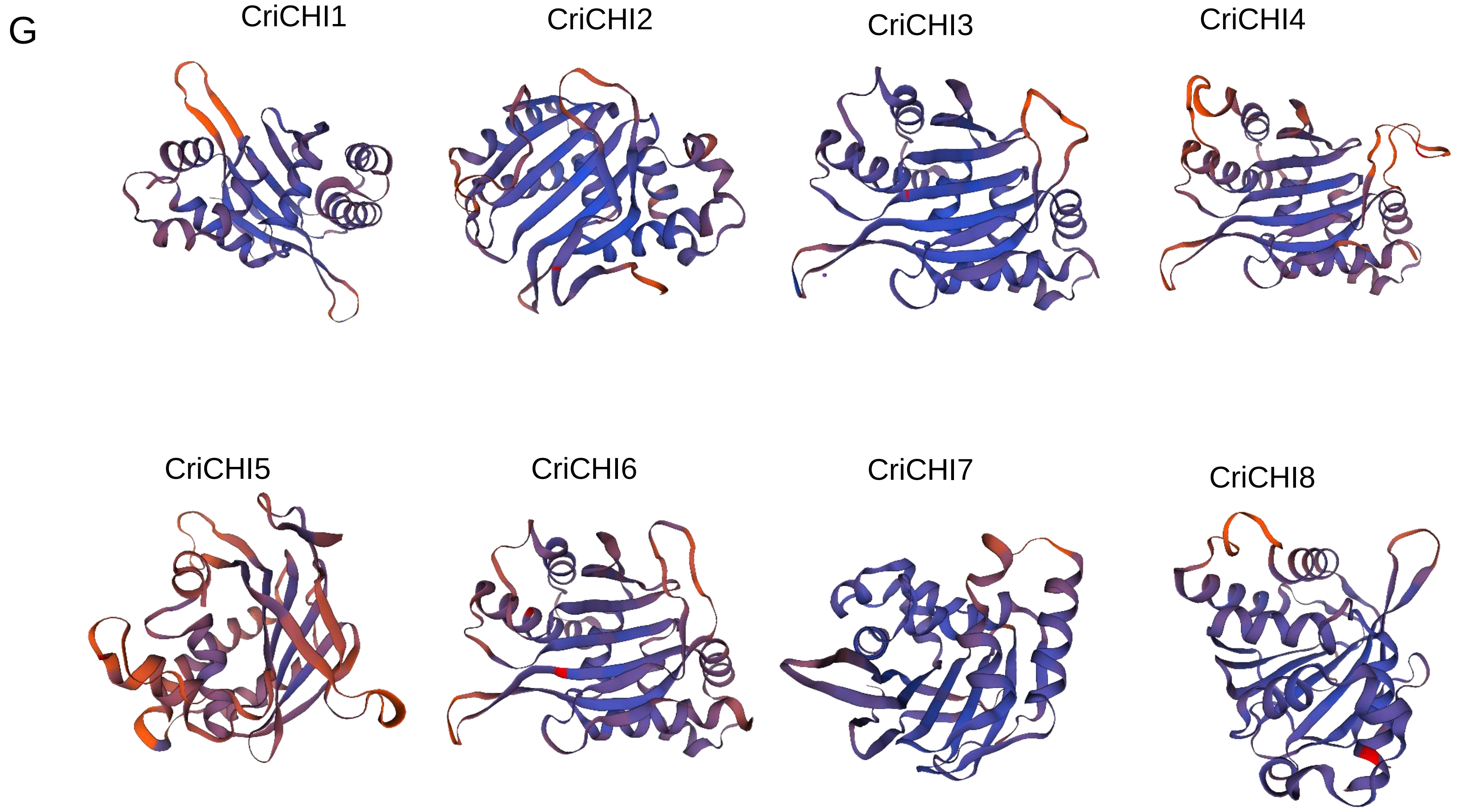
G
CriCHI1
CriCHI2
CriCHI3
CriCHI4
CriCHI5
CriCHI6
CriCHI7
CriCHI8

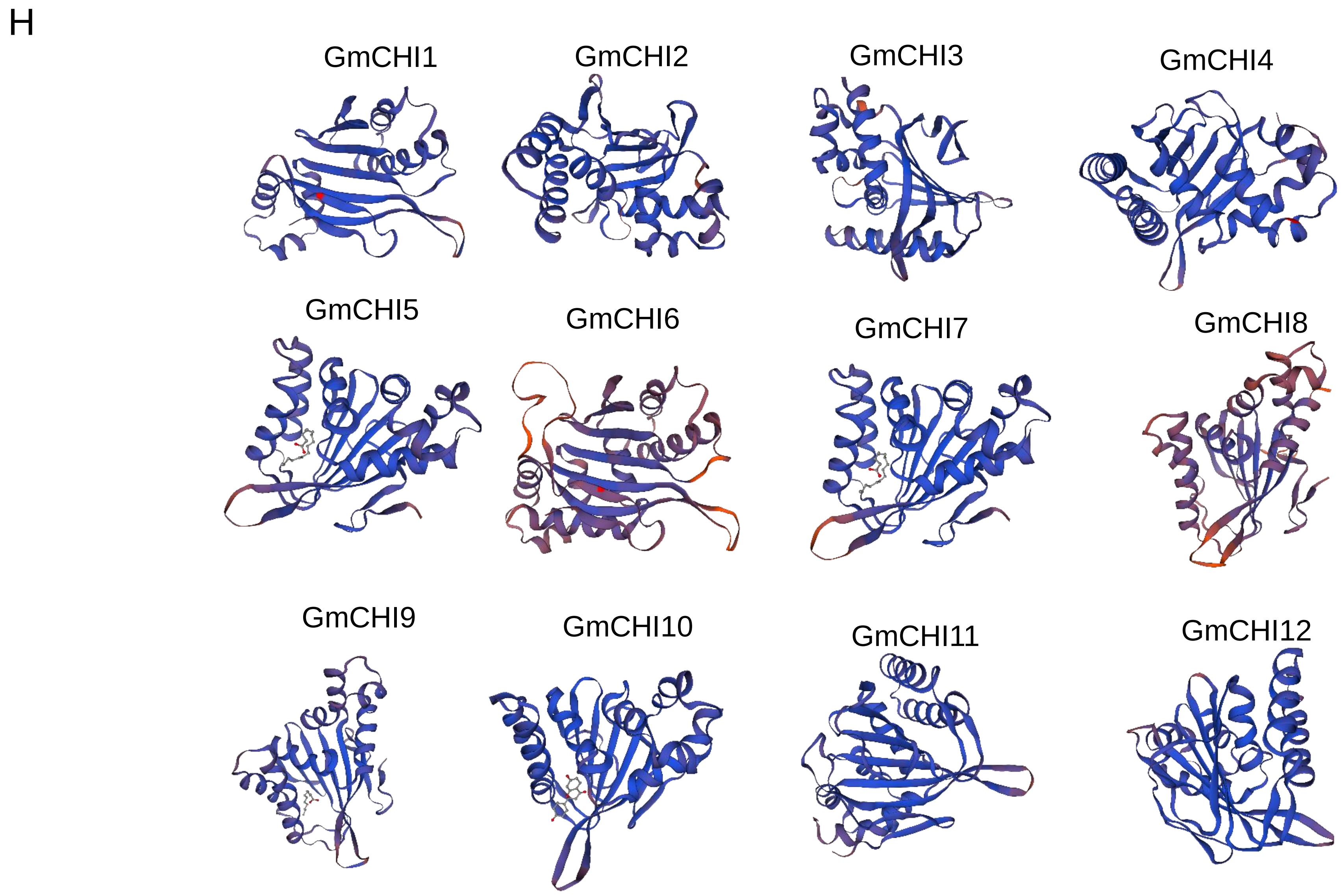
H
GmCHI1
GmCHI2
GmCHI3
GmCHI4
GmCHI5
GmCHI6
GmCHI7
GmCHI8
GmCHI9
GmCHI10
GmCHI11
GmCHI12

I

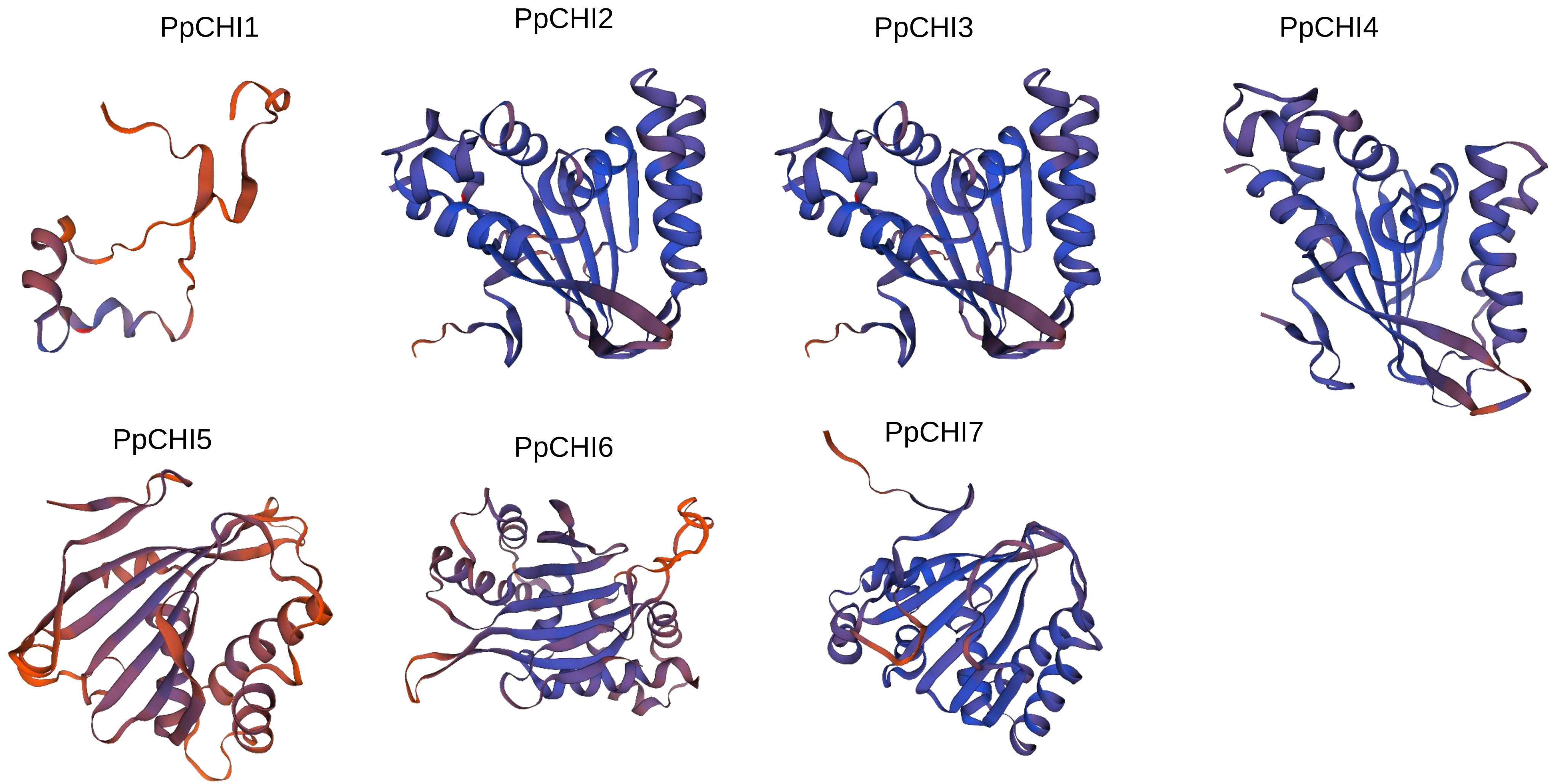

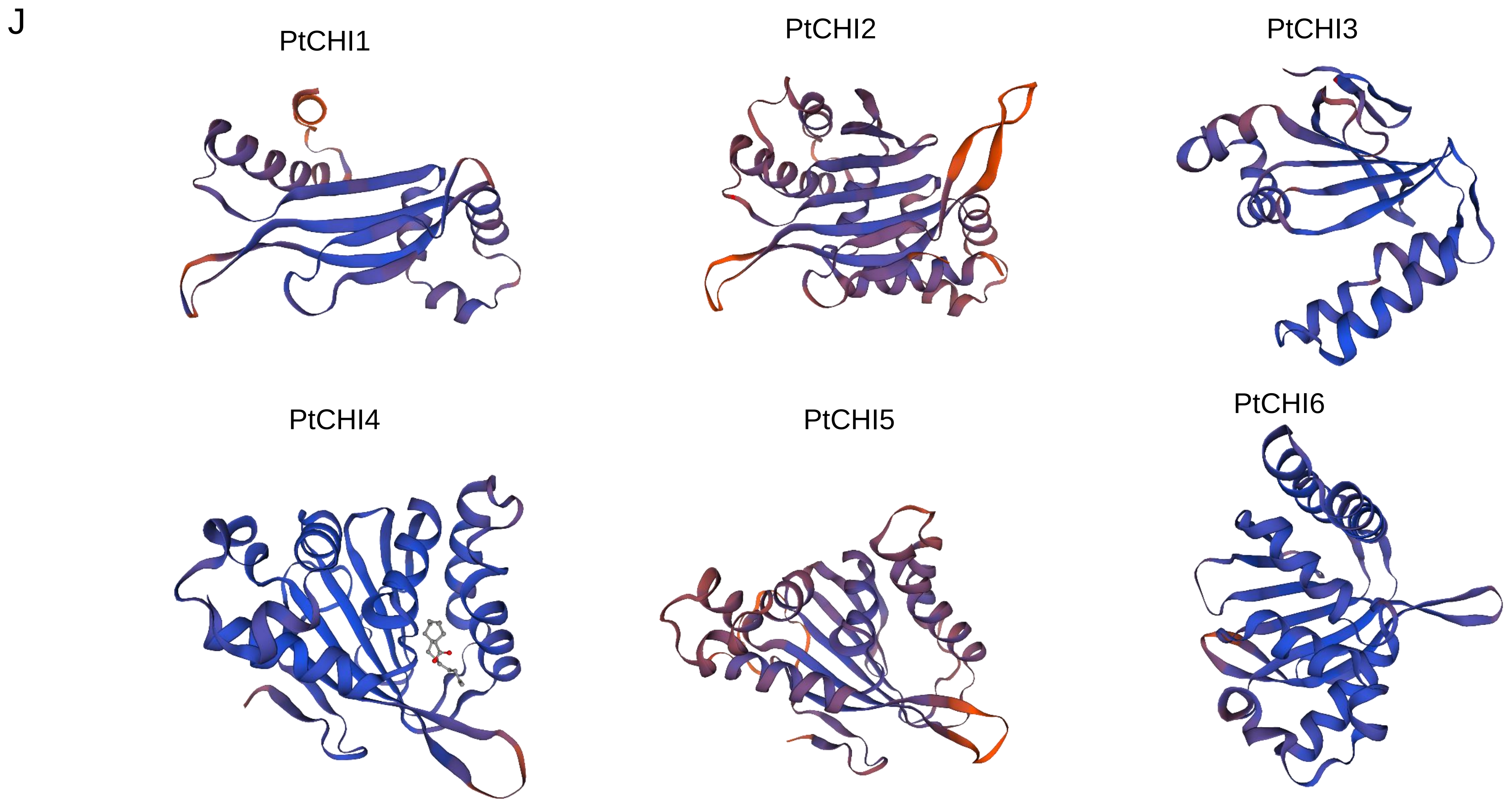
J
PtCHI1
PtCHI2
PtCHI3
PtCHI4
PtCHI5
PtCHI6

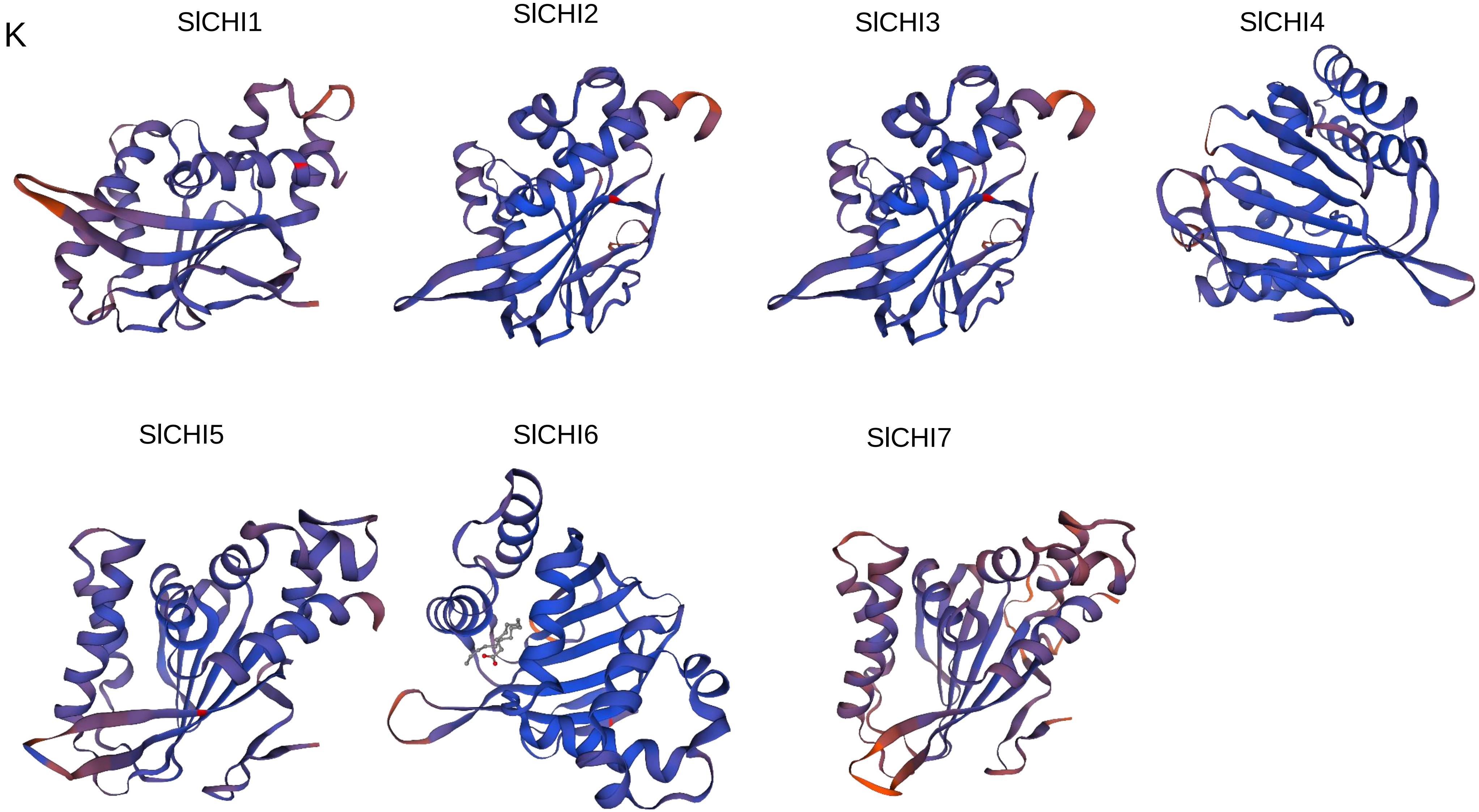
K
SlCHI1
SlCHI2
SlCHI3
SlCHI4
SlCHI5
SlCHI6
SlCHI7

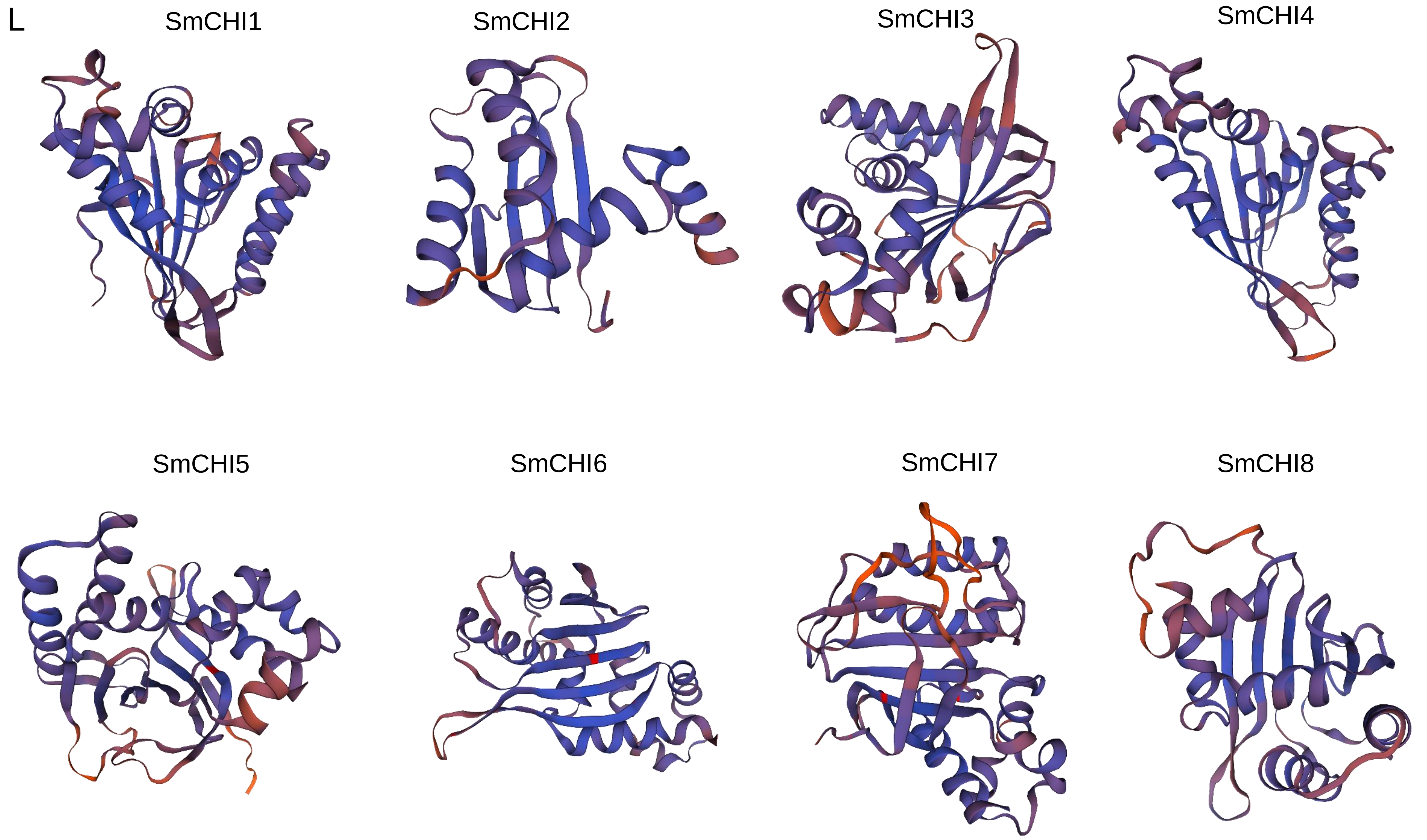
L
SmCHI1
SmCHI2
SmCHI3
SmCHI4
SmCHI5
SmCHI6
SmCHI7
SmCHI8

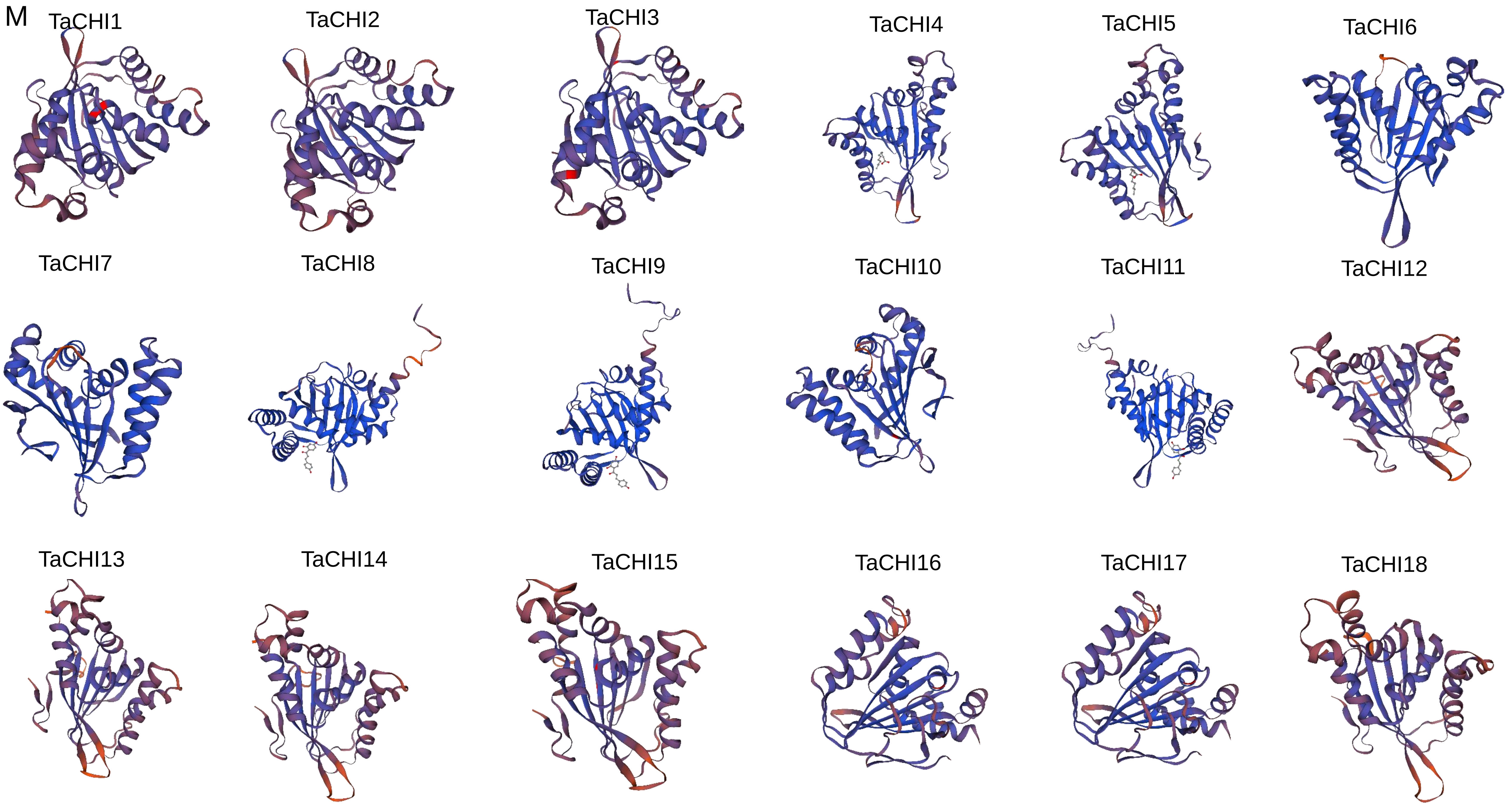
M
TaCHI1
TaCHI2
TaCHI3
TaCHI4
TaCHI5
TaCHI6
TaCHI7
TaCHI8
TaCHI9
TaCHI10
TaCHI11
TaCHI12
TaCHI13
TaCHI14
TaCHI15
TaCHI16
TaCHI17
TaCHI18

N

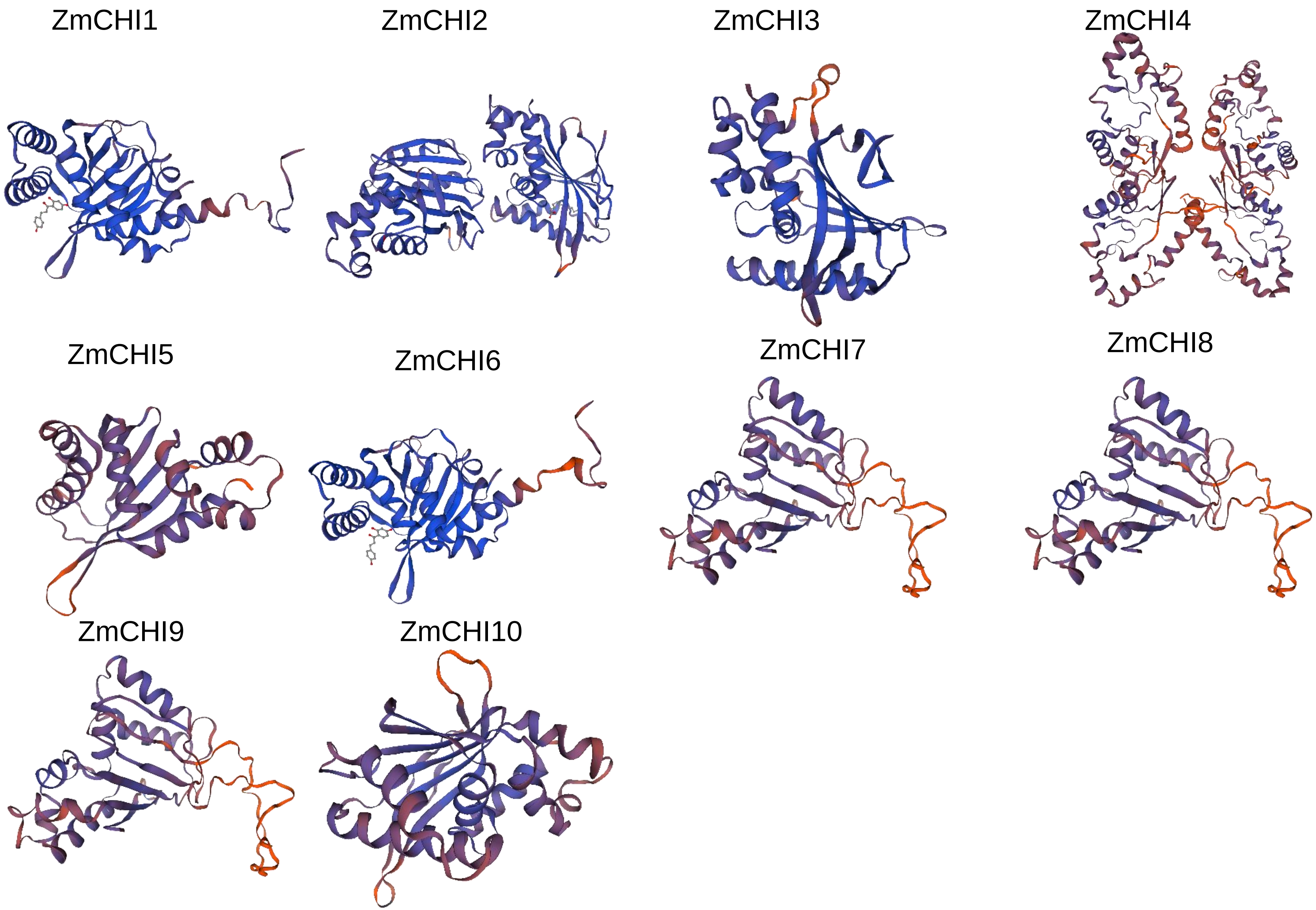
ZmCHI1
ZmCHI2
ZmCHI3
ZmCHI4
ZmCHI5
ZmCHI6
ZmCHI7
ZmCHI8
ZmCHI9
ZmCHI10

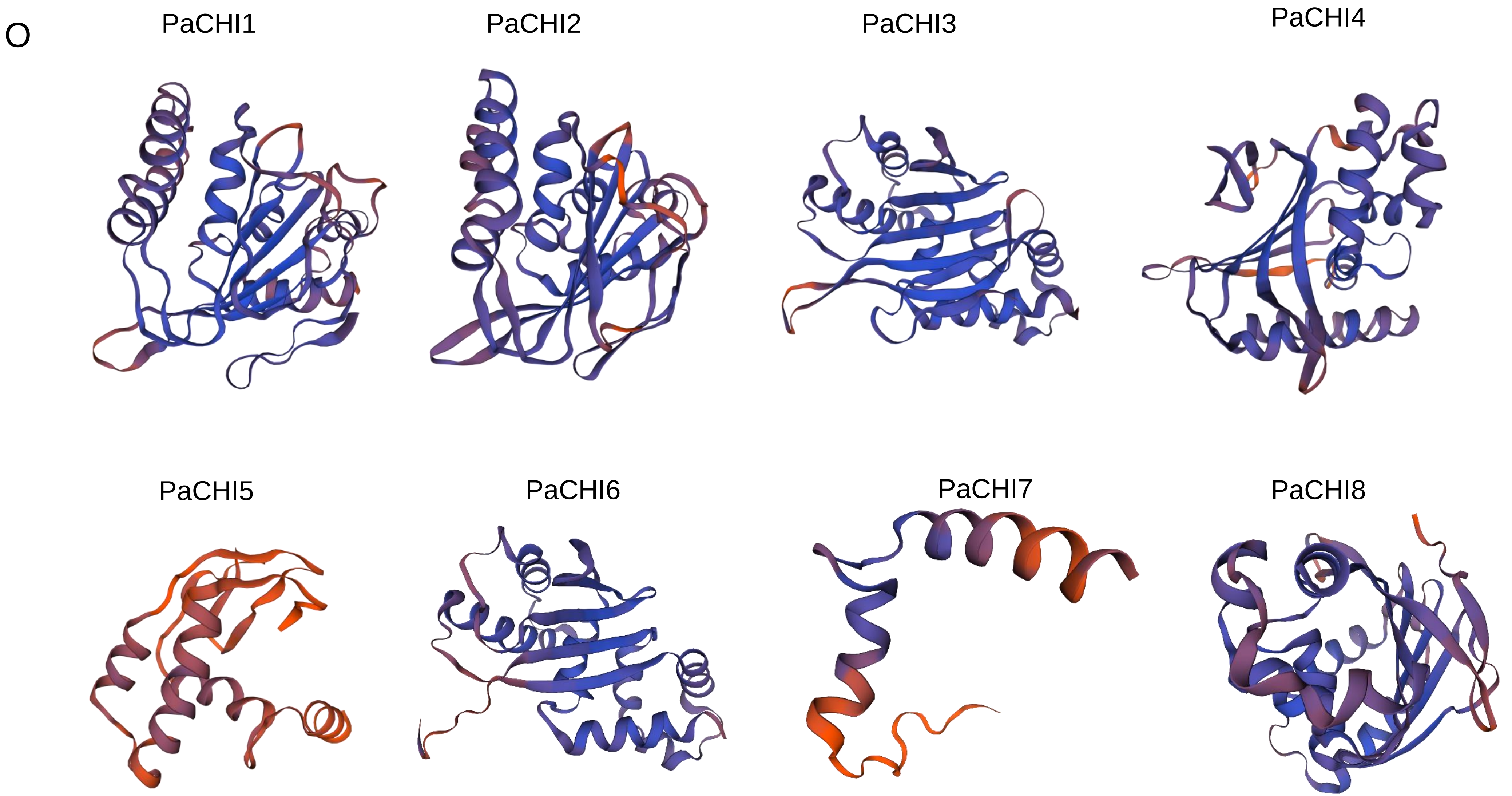

Supplementary Figure S4. 3D homology model of 122 CHIs among 15 green plants. A: *O.sativa*. B: *A.thaliana*. C: *C.reinhardtii*. D: *C.sativa*. E: *B.oleracea*. F: *C.quinoa*. G: *C.richardii*. H: *G.max*. I: *P .patens*. J: *P .trichocarpa*. K: *S.lycopersicum*. L: *S.moellendorffii*. M: *T.aestivum*. N: *Z.mays*. O: *P .abies*. The model was built using SWISS-MODEL. Blue colors represent α-helices and red colors represent β-stranded sheet. α-helices and β-stranded sheets connected by loops. Ball and stick represent the ligands.
