# Supplementary material for: Genome-Wide Classification and Evolutionary Analysis Reveal Diverged Patterns of Chalcone Isomerase in Plants
Source: Biomolecules. 2022 Jul 8;12(7):961. doi: 10.3390/biom12070961 (PMC9313115; doi:10.3390/biom12070961)
Supplement: Supplementary file 1 [file biomolecules-12-00961-s001.zip › Supplementary Figure caption.pdf]

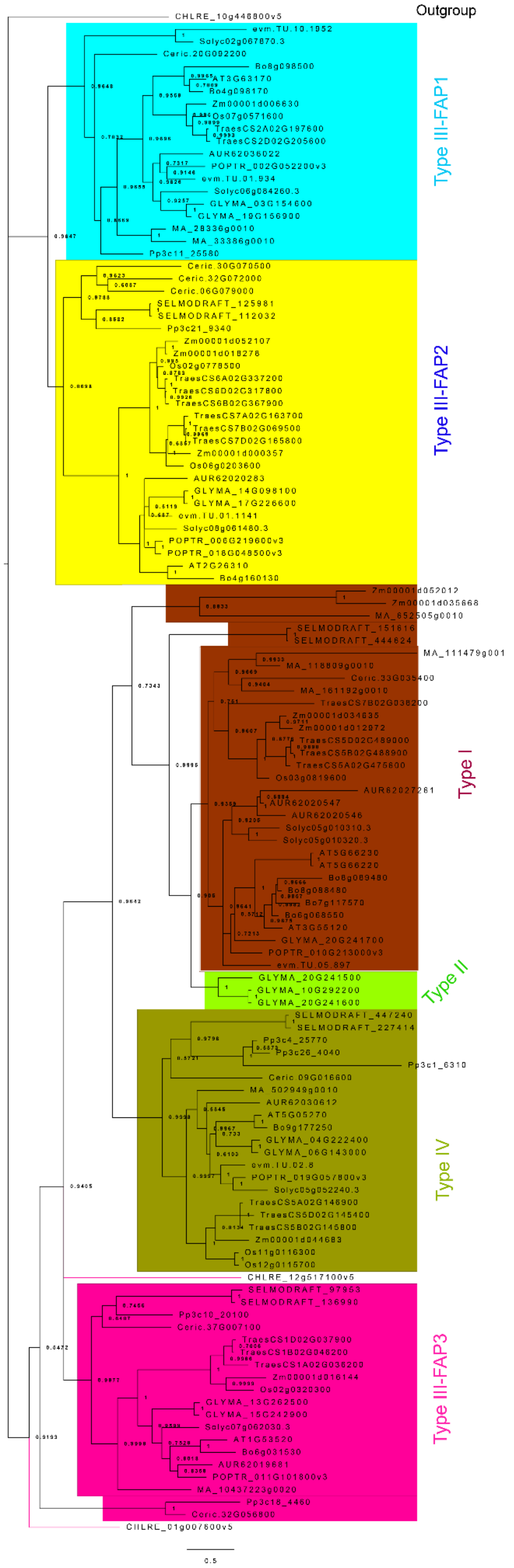

Figure S1. A Phylogenetic tree of green plant *CHI* genes was constructed using Bayesian method [46]. Different colors represent distinct lineages. FAP: fatty acid binding protein.

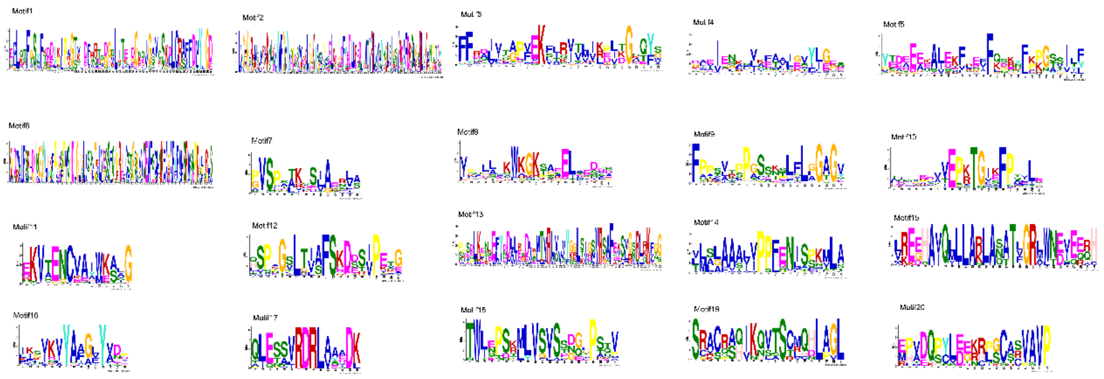

Figure S2. 20 putative conserved motifs were identified using MEME suite in this study (https://meme-suite.org/meme/tools/meme, accessed on 16 April 2022) [49].

Figure S3. Protein sequence alignment of CHIs among 15 green plants was shown by using ESPript 3.0 online tool (https://espript.ibcp.fr/ESPript/cgi-bin/ESPript.cgi, accessed on 8 April 2022) [43].The first line indicated the model protein sequence of MsCHI. The critical catalytic residues essential for bona fide CHI active sites are shown with red asterisks. Residues for the hydrogen bond network are marked with green asterisks. The blue boxes indicated the residues responsible for substrate preference.

Figure S4. 3D homology model of 122 CHIs among 15 green plants. A: *O.sativa.* B: *A.thaliana.* C: *C.reinhardtii.* D: *C.sativa.* E: *B.oleracea.* F: *C.quinoa.* G: *C.richardii.* H: *G.max.* I: *P .patens.* J: *P .trichocarpa.* K: *S.lycopersicum.* L: *S.moellendorffii.* M: *T.aestivum.* N: *Z.mays.* O: *P .abies.* The model was built using SWISS-MODEL server with default parameters (https://swissmodel.expasy.org/, accessed on 4 March 2022) [51]. Blue colors represent α-helices and red colors represent β-stranded sheet. α-helices and β-stranded sheets connected by loops. Ball and stick represent the ligands.

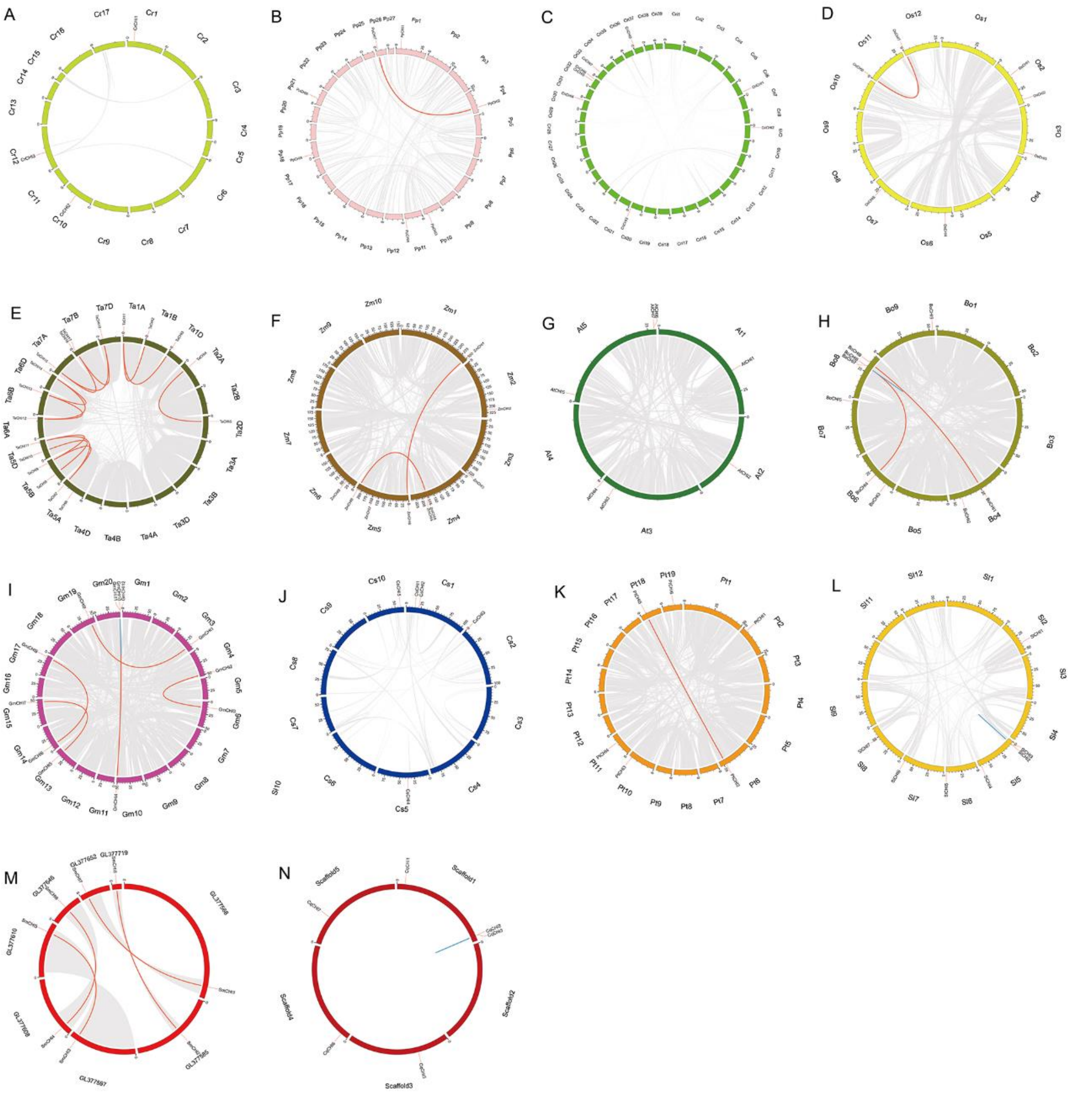

Figure S5. The chromosomal location and duplication events of *CHI* genes in 14 green plants. The circle plot was created by Circos software [56]. The chromosomes in each plant were indicated in different colors. Os, Zm, Ta, At, Bo, Cq, Cr, Cs, Gm, Pp, Pt, Sl, Sm, and Cri represented *O.sativa*, *Z.mays*, *T.aestivum*, *A.thaliana*, *B.oleracea*, *C.quinoa*, *C.reinhardtii*, *C.sativa*, *G.max*, *P.patens*, *P.trichocarpa*, *S.lycopersicum*, *S.moellendorffii* and *C.richardi* respectively. The red and blue lines separately represent segmental duplication and tandem duplication events. In addition, *CHIs* identified from *C.quinoa* and in *S.moellendorffii* could not be mapped to any chromosome conclusively, thus only the scaffolds containing *CHI* genes were used to analyze.

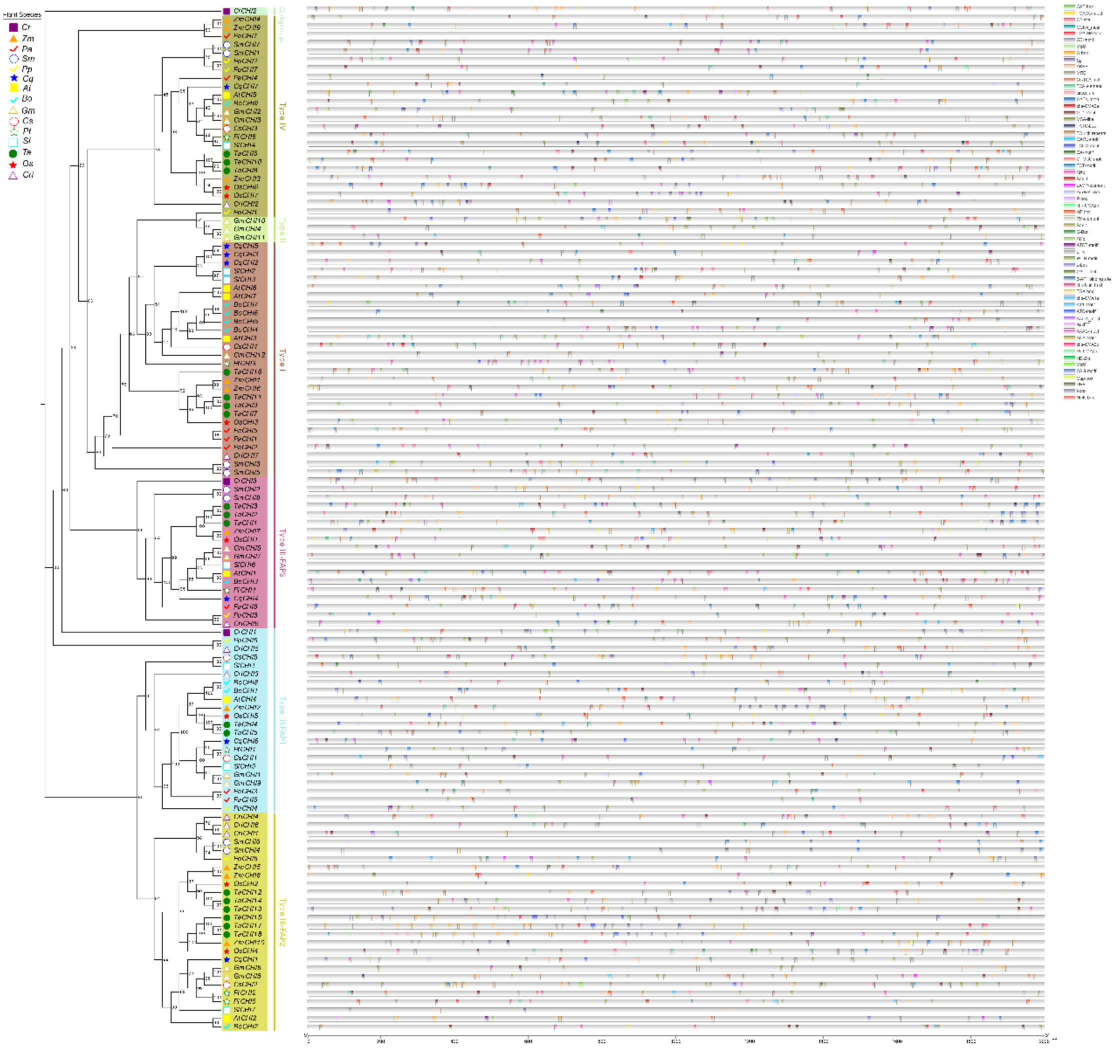

Figure S6. *Cis*-acting elements of all *CHI* genes were shown based on the phylogenetic tree. The maximum likelihood (ML) phylogenetic tree was developed by using the IQ-TREE tool with 1000 bootstrap replicates [45]. Details of classifications were shown in different colors. FAP: fatty acid binding protein. Os, Zm, Ta, At, Bo, Cq, Cr, Cs, Gm, Pp, Pt, Sl, Sm, and Cri represented *O.sativa*, *Z.mays*, *T.aestivum*, *A.thaliana*, *B.oleracea*, *C.quinoa*, *C.reinhardtii*, *C.sativa*, *G.max*, *P.patens*, *P.trichocarpa*, *S.lycopersicum*, *S.moellendorffii* and *C.richardi* respectively. Different promoter elements in each *CHI* gene were indicated by different colored boxes.

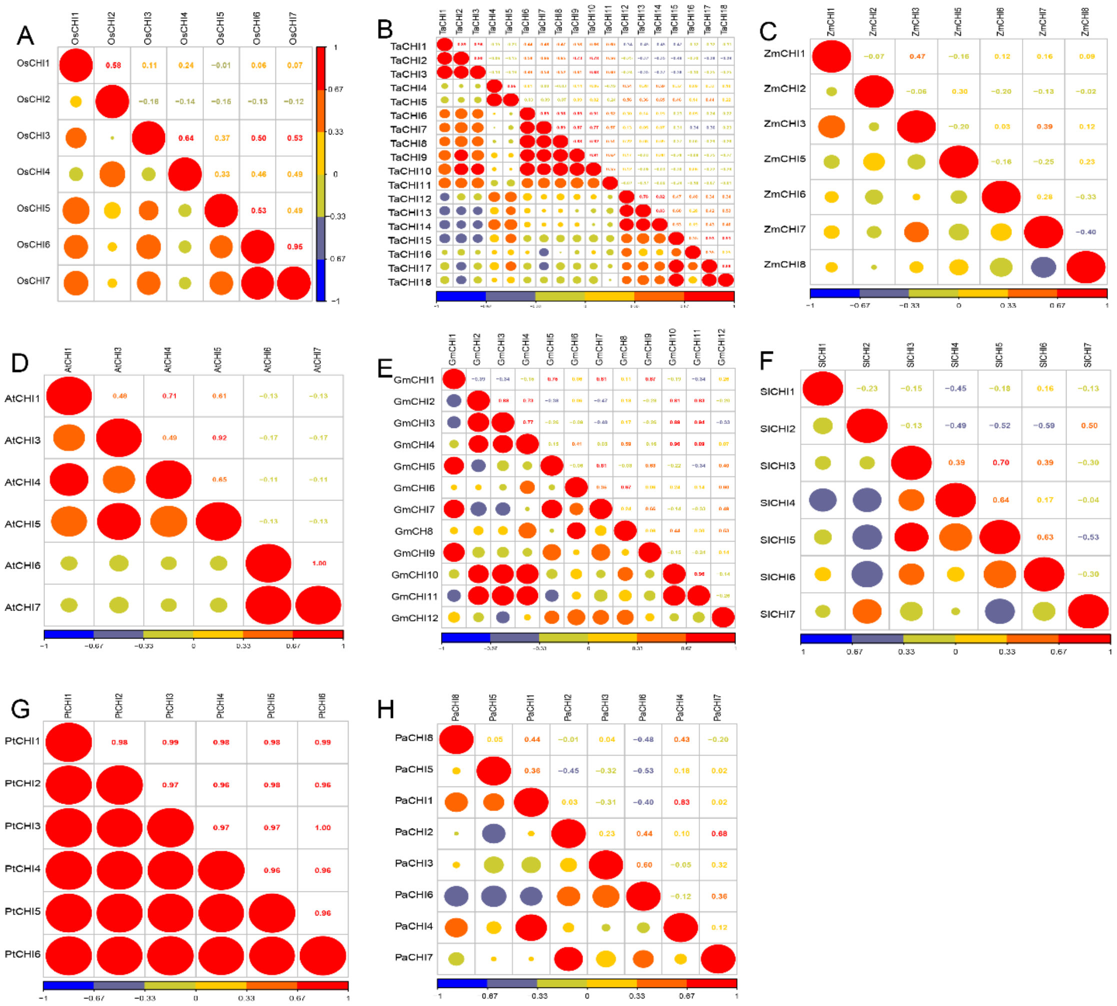

Figure S7. Pearson's correlation coefficient between CHI genes in individual plants at the transcriptional level among 8 green plants, including *O.sativa* (**A**), *T.aestivum* (**B**), *Z.mays* (**C**), *A.thaliana* (**D**), *G.max* (**E**), *S.lycopersicum* (**F**), *P.trichocarpa* (**G**), and *P.abies* (**H**), respectively.
