# Supplementary figures and images for: Genome-Wide Classification and Evolutionary Analysis Reveal Diverged Patterns of Chalcone Isomerase in Plants
Source: Biomolecules. 2022 Jul 8;12(7):961. doi: 10.3390/biom12070961 (PMC9313115; doi:10.3390/biom12070961)

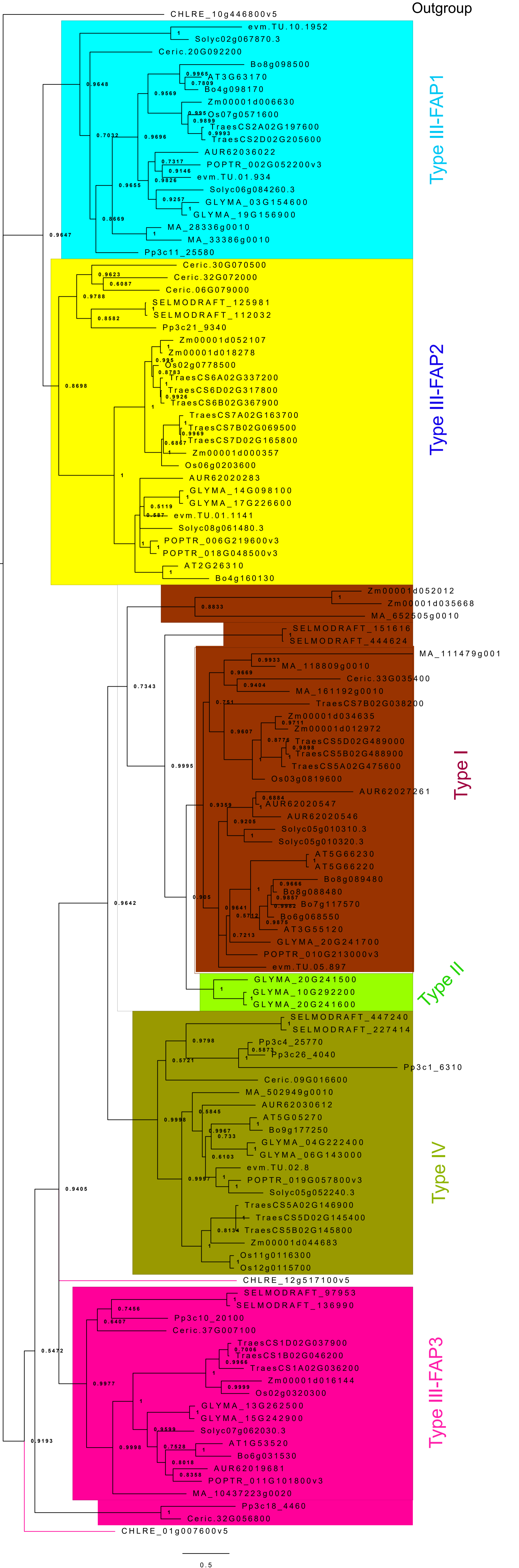

0.5

Supplement: Supplementary file 1 [file biomolecules-12-00961-s001.zip › Figure S1 A Phylogenetic tree of green plant CHI genes was constructed using Bayesian method.pdf]

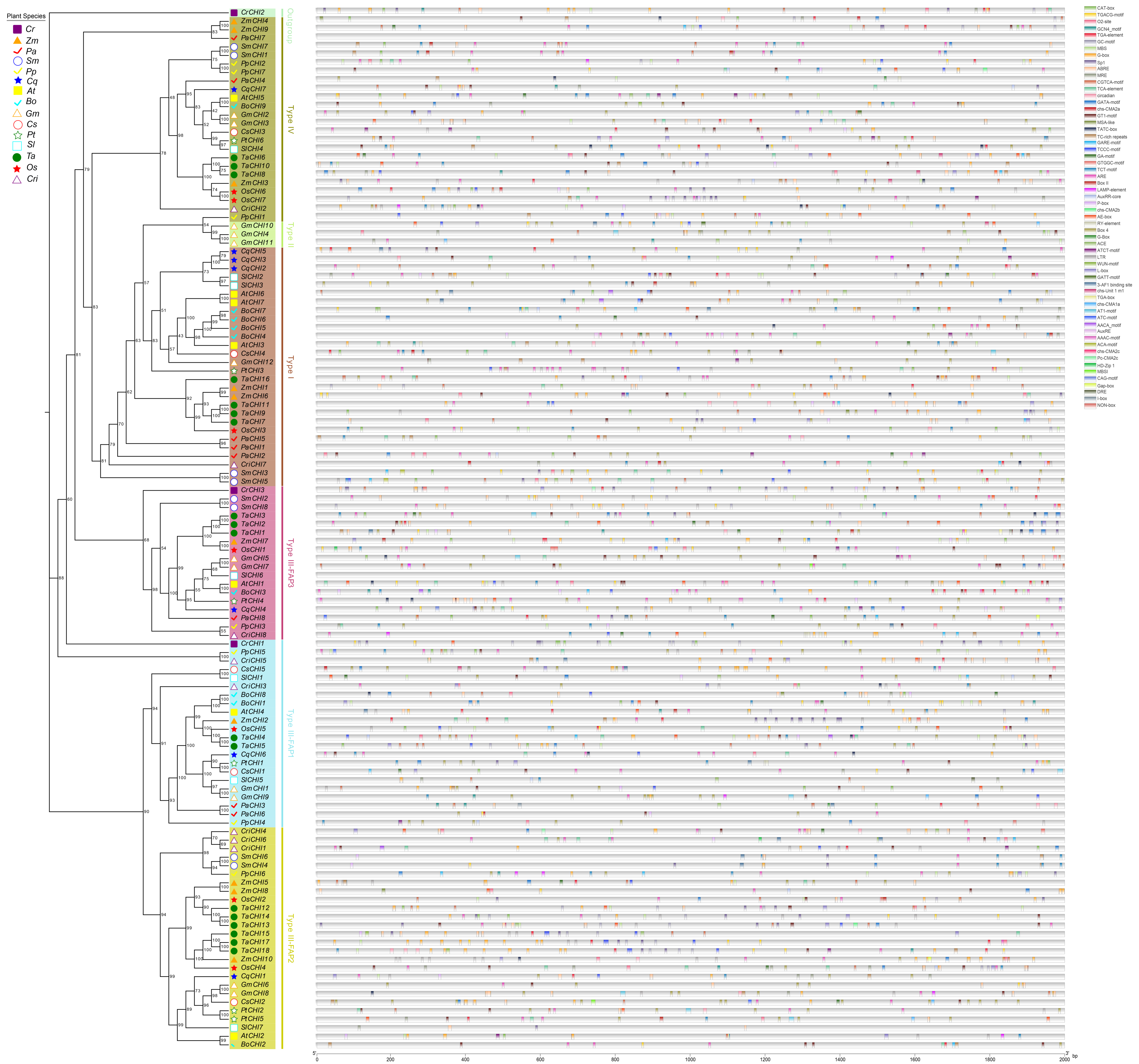

Supplement: Supplementary file 1 [file biomolecules-12-00961-s001.zip › Figure S6 Cis-acting elements of all CHI genes were shown based on the phylogenetic tree.pdf]

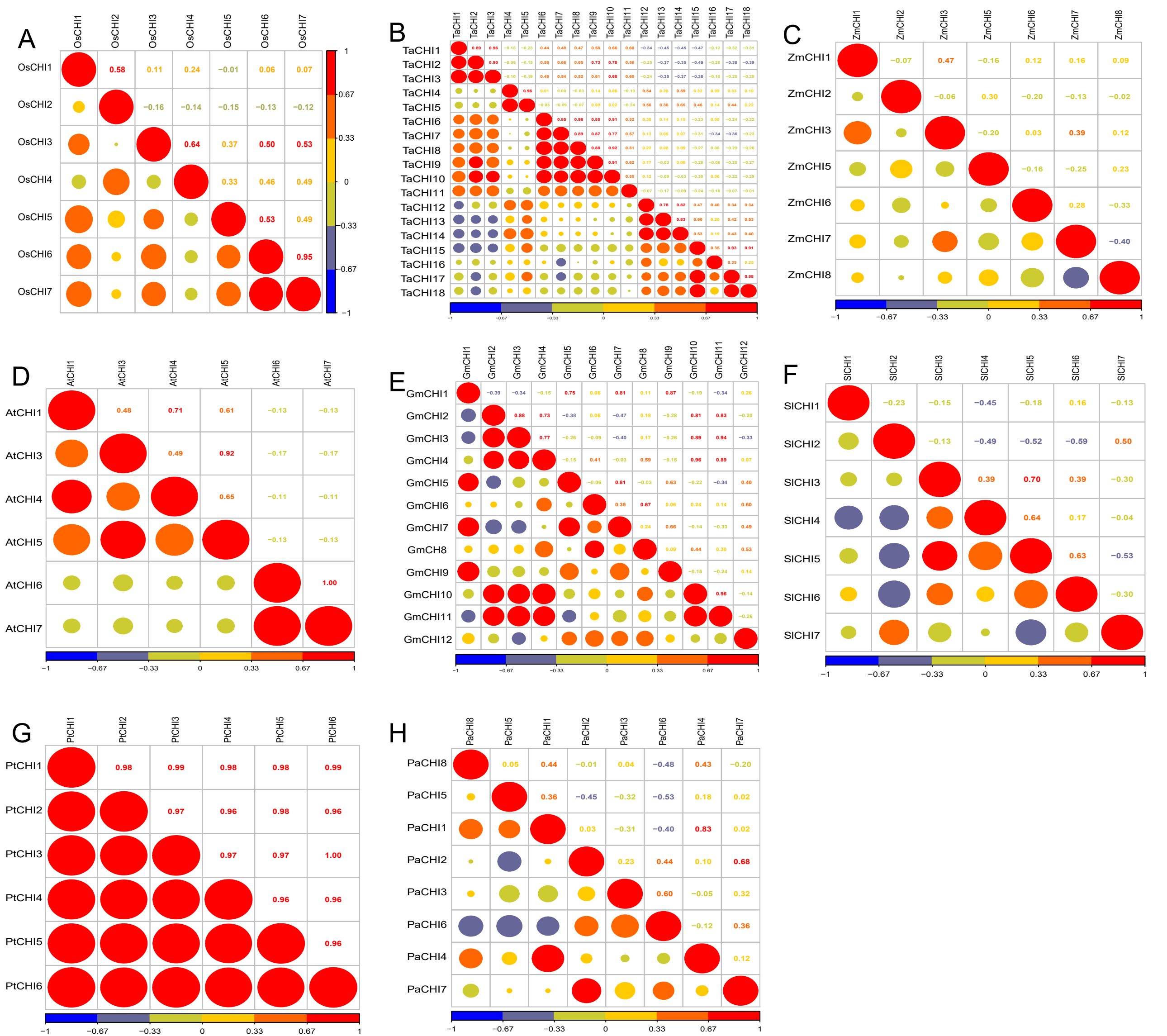

Supplement: Supplementary file 1 [file biomolecules-12-00961-s001.zip › Figure S7. Pearsoní»s correlation coefficient among CHI genes at the transcriptional level among 8 green plants..pdf]
