# Supplementary material for: Genome-Wide Classification and Evolutionary Analysis Reveal Diverged Patterns of Chalcone Isomerase in Plants
Source: Biomolecules. 2022 Jul 8;12(7):961. doi: 10.3390/biom12070961 (PMC9313115; doi:10.3390/biom12070961)
Supplement: Supplementary file 1 [file Figure S5. The chromosomal location and duplication events of CHI genes in 14 green plants.pdf › Figure S5. The chromosomal location and duplication events of CHI genes in 14 green plants.pdf]

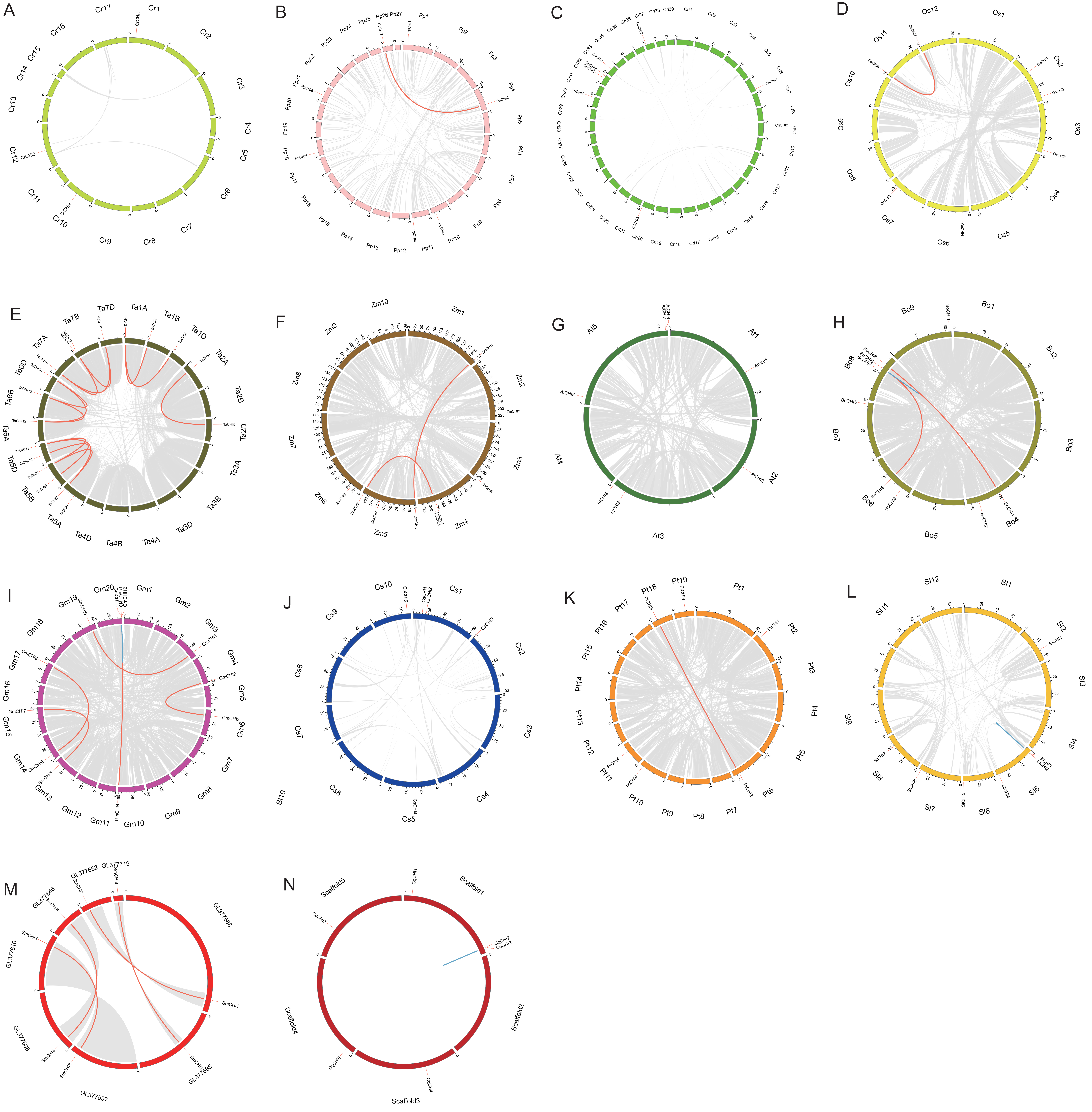
A
Cr1
Cr2
Cr3
Cr4
Cr5
Cr6
Cr7
Cr8
Cr9
Cr10
Cr11
Cr12
Cr13
Cr14
Cr15
Cr16
Cr17
B
Pp1
Pp2
Pp3
Pp4
Pp5
Pp6
Pp7
Pp8
Pp9
Pp10
Pp11
Pp12
Pp13
Pp14
Pp15
Pp16
Pp17
Pp18
Pp19
Pp20
Pp21
Pp22
Pp23
Pp24
Pp25
Pp26
Pp27
C
D
Os1
Os2
Os3
Os4
Os5
Os6
Os7
Os8
Os9
Os10
Os11
Os12
E
Ta1A
Ta1B
Ta1D
Ta2A
Ta2B
Ta2D
Ta3A
Ta3B
Ta3D
Ta4A
Ta4B
Ta4D
Ta5A
Ta5B
Ta5D
Ta6A
Ta6B
Ta6D
Ta7A
Ta7B
Ta7D
F
Zm1
Zm2
Zm3
Zm4
Zm5
Zm6
Zm7
Zm8
Zm9
Zm10
G
At1
At2
At3
At4
At5
H
Bo1
Bo2
Bo3
Bo4
Bo5
Bo6
Bo7
Bo8
Bo9
I
Gm1
Gm2
Gm3
Gm4
Gm5
Gm6
Gm7
Gm8
Gm9
Gm10
Gm11
Gm12
Gm13
Gm14
Gm15
Gm16
Gm17
Gm18
Gm19
Gm20
J
Cs1
Cs2
Cs3
Cs4
Cs5
Cs6
Cs7
Cs8
Cs9
Cs10
K
Pt1
Pt2
Pt3
Pt4
Pt5
Pt6
Pt7
Pt8
Pt9
Pt10
Pt11
Pt12
Pt13
Pt14
Pt15
Pt16
Pt17
Pt18
Pt19
L
Sl1
Sl2
Sl3
Sl4
Sl5
Sl6
Sl7
Sl8
Sl9
Sl10
Sl11
Sl12
M
GL377568
GL377585
GL377597
GL377608
GL377610
GL377646
GL377652
GL377719
N
Scaffold1
Scaffold2
Scaffold3
Scaffold4
Scaffold5
